# Multi-timescale Rhythmic Dynamics in Rostral Ventromedial Medulla Neurons

**DOI:** 10.64898/2025.12.15.693905

**Published:** 2025-12-17

**Authors:** Carl Ashworth, Melissa Martenson, Zhigang Shi, Caitlynn C. De Preter, Mary Heinricher, Flavia Mancini

**Affiliations:** 1 Computational and Biological Learning Lab, Department of Engineering, University of Cambridge, Cambridge, United Kingdom; 2 Department of Neurological Surgery, Oregon Health & Science University, Portland, United States of America; 3 Department of Behavioral Neuroscience, Oregon Health & Science University, Portland, United States of America; 4 Department of Biomedical Engineering, Oregon Health & Science University, Portland, United States of America

## Abstract

Neural circuits involved in sensory control must integrate fast, reflexive responses with slower, state-dependent modulation. This coordination is especially critical in the pain system, where rapid withdrawal must be balanced against slower adjustments in nociceptive sensitivity that reflect arousal, physiology, and internal state. We hypothesized that neurons of the rostral ventromedial medulla (RVM)—the principal brainstem node for descending pain control—operate across multiple temporal scales, combining fast stimulus-evoked responses with slower intrinsic dynamics. Using single-unit recordings from identified RVM ON-, OFF-, and NEUTRAL-cells and probabilistic modeling, we find that ON- and OFF-cells exhibit multi-phase population responses characterized by rapid activation and prolonged recovery dynamics lasting tens of seconds. In the absence of stimulation, the same neurons display coherent, quasi-periodic oscillations on the order of minutes, captured by Gaussian process models as predictable, low-frequency fluctuations. These rhythmic dynamics were specific to ON- and OFF-cells and occasionally coherent with autonomic parameters, suggesting coupling between nociceptive and homeostatic control loops. Together, these results identify a multi-timescale organizational principle within descending brainstem circuits, in which fast, reflex-related and slow, rhythmic processes jointly structure sensory modulation and internal state control.

Neural systems that regulate sensory processing must operate across multiple timescales. Reflexive responses unfold within hundreds of milliseconds, yet sensory gain and behavioral sensitivity fluctuate over seconds to minutes according to arousal, physiology, and internal state. How these distinct temporal regimes are coordinated within modulatory pathways that shape sensory processing remains largely unknown. In the nociceptive system, this question is particularly pressing: effective pain control must balance rapid withdrawal reflexes with slower, state-dependent adjustments in nociceptive sensitivity that support adaptive behavior. The rostral ventromedial medulla (RVM) is a key node in a descending modulatory pathway that regulates nociceptive processing at the level of the dorsal horn^1,2^. Through direct projections to the spinal dorsal horn, the RVM exerts potent inhibitory and facilitatory control over nociceptive transmission^3,4^. Its ON- and OFF-cells are classically defined by their reciprocal responses to noxious stimulation^5^ (Figure 1): ON-cells increase firing before a nocifensive withdrawal, while OFF-cells pause. RVM cells that do not appear to be modulated by noxious stimuli are generically classed as NEUTRAL-cells. These dynamics have traditionally been viewed as reflex-linked, transient responses that shape the timing of motor output.

Yet several features of RVM organization suggest a broader and more active role. The RVM is positioned to integrate both bottom-up sensory drive from the spinal cord and sustained top-down control from the periaqueductal gray, hypothalamus, and other homeostatic centers^6–8^, and it participates in thermoregulatory and autonomic control^9–11^. Work comparing direct versus indirect recruitment of spinal and trigeminal inputs through the parabrachial complex further indicates that RVM neurons can exhibit both transient and prolonged responses, depending on the route of activation—hinting at an intrinsic capacity to integrate nociceptive signals across distinct timescales^12^. Moreover, early single-unit recordings hinted that ON- and OFF-cells may show coordinated or reciprocal fluctuations even in the absence of sensory stimulation^13^. However, these observations were limited in scope, and the temporal organization of both evoked and ongoing RVM activity has never been quantitatively defined. As a result, it has remained unclear whether the RVM functions primarily as a reactive relay or as a dynamic controller operating across multiple temporal domains.

Several gaps therefore remain in our understanding of RVM function. First, the temporal structure of the ON- and OFF-cell response has not been quantitatively characterized beyond the brief epoch surrounding withdrawal, leaving open the question of how these responses evolve and recover over time. Second, the ongoing activity of RVM neurons under unstimulated conditions has been largely unexplored, despite the fact that descending modulation is continuously engaged in vivo. Third, it is unknown whether slow fluctuations in RVM activity relate to physiological variables such as autonomic rhythms, given the RVM’s role at the intersection of nociceptive and homeostatic control.

Here we combine single-unit recordings from identified ON-, OFF-, and NEUTRAL-cells with probabilistic modeling to address these gaps. We asked two main questions: (1) How do ON- and OFF-cell firing rates evolve during and after noxious stimulation, and do these dynamics reveal distinct fast and slow components of descending control? (2) Do RVM neurons exhibit structured temporal organization during ongoing, unstimulated activity?

We hypothesized that RVM neurons operate across multiple temporal scales: that ON- and OFF-cells would exhibit fast, asymmetric response–recovery dynamics linked to reflex control, and slower, internally generated oscillations reflecting network or state-dependent modulation. Using Bayesian and Gaussian process modeling, we find strong evidence for both. RVM neurons express multi-timescale population dynamics—fast, multi-phase responses to noxious stimuli unfolding over seconds to tens of seconds, and slow, rhythmic fluctuations in ongoing activity occurring over minutes. These oscillations, specific to ON- and OFF-cells, were predictable from past activity and occasionally coherent with autonomic rhythms, suggesting coupling between nociceptive and homeostatic control loops. Together, these findings reveal a hierarchical temporal organization within descending pain pathways, in which fast reflex-related and slow rhythmic processes jointly structure sensory modulation and internal state control.

## Results

### Canonical ON/OFF responses to noxious stimulation

**Figure 1:**
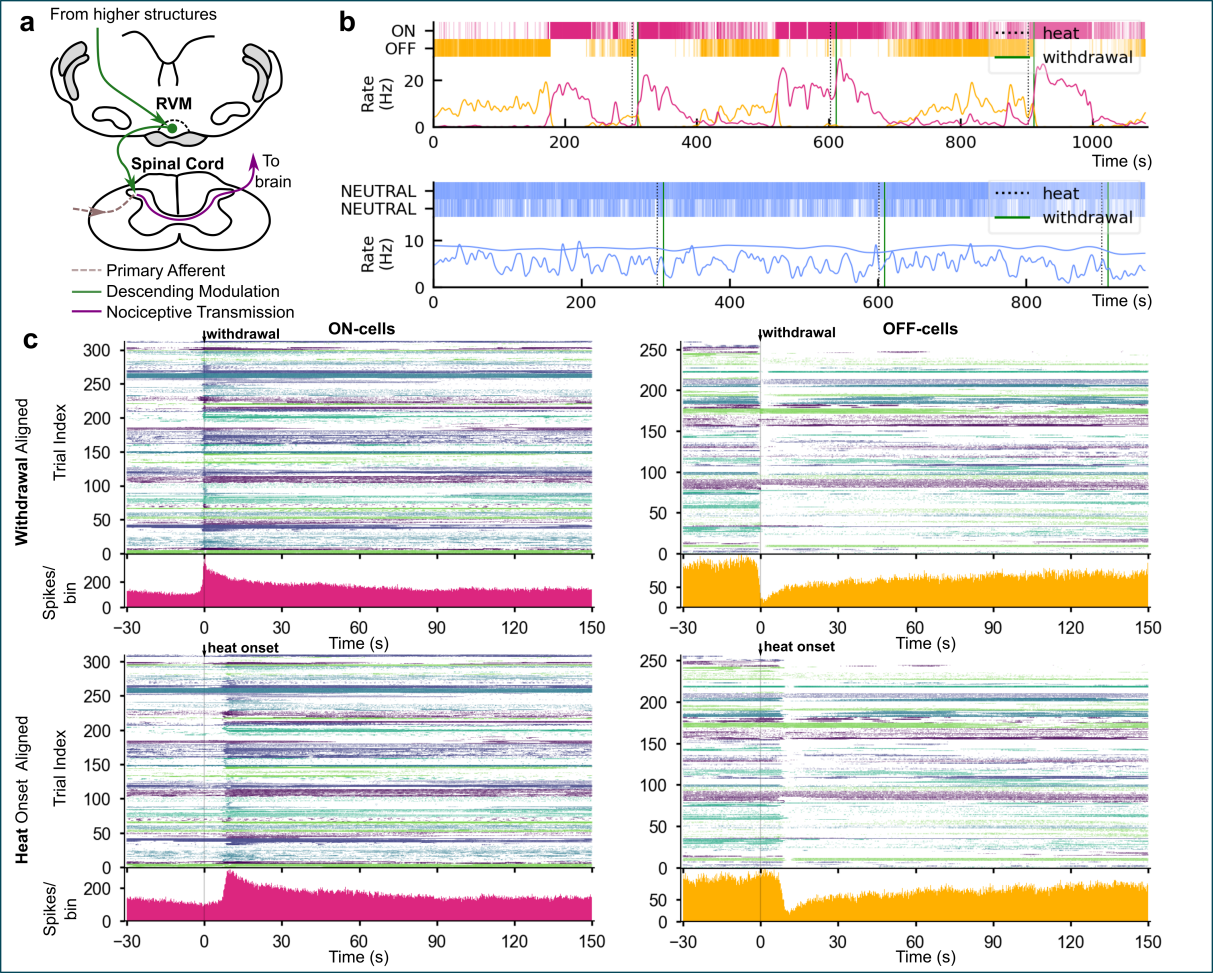
Stimulation-evoked activity of ON-, OFF-, and NEUTRAL-cells in the RVM. **a**, Schematic of the RVM and its descending projections to the spinal dorsal horn, illustrating the modulatory roles of ON- and OFF-cells in nociceptive processing. **b**, Representative spike rasters (top) and rate plots (bottom) from simultaneously recorded ON-, OFF-, and NEUTRAL-cells during repeated noxious heat trials. ON- and OFF-cells exhibit the canonical reciprocal dynamics around paw withdrawal (solid green line), whereas NEUTRAL-cells show no clear modulation. Notably, alternating dominance of ON- and OFF-cells is also observed during inter-trial intervals, suggesting ongoing, stimulus-independent dynamics. **c**, Population peri-stimulus time histograms (PSTHs) for ON- and OFF-cells, aligned either to the time of paw withdrawal (top) or to the heat stimulus onset (bottom). Population firing is sharply time-locked to the withdrawal reflex but not to the onset of heat application. Withdrawal-aligned PSTHs show steeper slopes and no delay compared to heat-aligned PSTHs, consistent with the nocifensive response, rather than the stimulus as such, being the primary driver of RVM activity.

To validate cell classification, we first confirmed that ON- and OFF-cells exhibited canonical reciprocal dynamics during noxious heat stimulation^5,13–16^. Figure 1b shows an example ON- (pink) and OFF-cell (yellow) pairing recorded simultaneously in one animal, and two NEUTRAL-cells (blue) recorded in a second animal, each shown as both raster and firing rate plots. During noxious heat stimulation (vertical, black dotted line), the ON-cell increased firing in close temporal proximity to the paw withdrawal, whereas the OFF-cell sharply decreased firing, consistent with canonical reciprocal dynamics. Notably, spontaneous switches between OFF- and ON-dominant activity were also observed during intertrial intervals (e.g., at 180 s and 520 s), indicating that activity of these cell classes is not solely stimulus-evoked but also reflects internal or ongoing network dynamics. In contrast, NEUTRAL-cells showed no change associated with the stimulus trials (see Supplementary Figure 3), consistent with their canonical lack of nociceptive responsiveness.

Population responses are summarized in Figure 1c, which shows peri-stimulus time histograms (PSTHs) for 70 ON-cells and 60 OFF-cells, aligned to the time of paw withdrawal (Figure 1c, upper) or to the onset of heat stimulation (Figure 1c, lower). In total, 314 ON-cell and 261 OFF-cell trials were aligned to withdrawal, while 311 ON-cell and 257 OFF-cell trials were aligned to heat onset (3 to 4 trials/cell, trial breakdown per animal is provided in the Supplementary Information). PSTHs showed a clear lag and shallower slopes when aligned to heat onset compared to withdrawal, confirming that our dataset captures the canonical, reciprocal ON/OFF responses associated with nocifensive reflexes.

Notably, ON- and OFF-cell activity also evolved on longer timescales that extended beyond the time of withdrawal, suggesting both fast and slow dynamics. We therefore modeled ON- and OFF-cell population dynamics using Bayesian regression to characterize these response and recovery timescales.

### Evoked ON/OFF activity unfolds over multiple timescales

**Figure 2:**
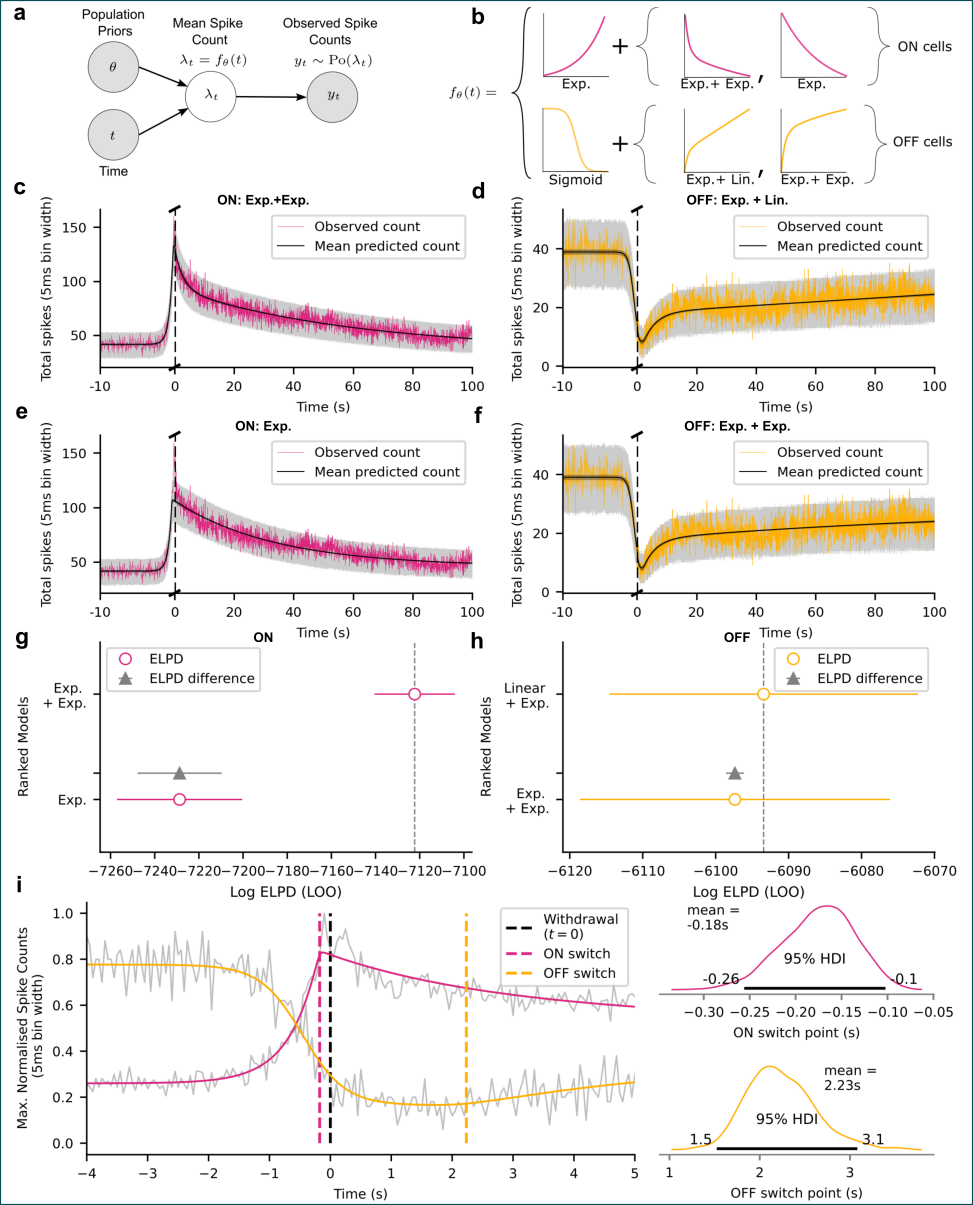
Bayesian modeling of ON- and OFF-cell population dynamics during noxious stimulation. **a**, The probabilistic model used to describe withdrawal-aligned population firing rates. **b**, Candidate response–recovery functions per cell type. For ON-cells, single vs. double exponential (exp.) recovery models were compared; for OFF-cells, double exp. vs. linear + exp. recovery models were evaluated. **c**-**f**, Models were fitted to the population firing rates per cell type and model variant. The single exp. recovery model missed the sharp ON-cell peak (**e**) and the double exp. model underestimated the OFF-cell recovery rate (**f**). **g**-**h**, Expected Log Predictive Density (ELPD) comparison of models, confirming that the double exp. recovery best fit ON-cells, while the linear + exp. model best fit OFF-cells. Whilst the linear + exp. model performed best for OFF-cells, the improvement over the double exp. model was minimal. **i**, Combined ON- and OFF-cell model fits around the withdrawal and population data in gray (left). Posterior switching timepoint estimates ($t_{s}$) per cell type (right), showing that ON-cells peak slightly before withdrawal, while OFF-cells exhibit post-withdrawal suppression.

To characterize the evoked response and recovery dynamics of ON- and OFF-cell populations, we applied a piecewise non-linear Bayesian regression model with a Poisson likelihood to the summed spike counts per time bin across the entire ON- and OFF-cell populations, aligned initially by withdrawal time (Figure 2a; see Methods). Cells were not distinguished by their firing rate pre-trial and all trials with a withdrawal were included. We focused on population-level structure rather than animal- or cell-specific effects, given the small number of trials per animal and the absence of a principled basis for differential weighting. Under the assumption of exchangeability, pooling across cells and animals reduces uncorrelated noise and preserves shared population dynamics. We used Bayesian highest density intervals (HDIs), defined as the minimum width Bayesian credible intervals^17^. A 97% HDI means there is a 97% probability that the unobserved parameter lies within the given interval, given the model and data.

For all analyses of evoked activity, we decomposed each trial into two segments: a response phase, defined as the monotonic departure of firing rate from the pre-withdrawal baseline (increase for ON-cells, decrease for OFF-cells), and a recovery phase, defined as the monotonic return toward a post-withdrawal baseline. These were separated by a single transition timepoint $t_{s}$, corresponding to the extremum (peak for ON-cells, nadir for OFF-cells) before recovery began. A null model assuming a constant mean spike count performed significantly worse than all piecewise models, confirming that both an evoked departure and a distinct recovery process were required to explain the data (see Supplementary Figure 8). Since all trials were aligned to withdrawal, variability in the withdrawal latency (time between heat onset and withdrawal) cannot shift post-withdrawal activity into the pre-withdrawal window, making an averaging artifact an unlikely source of the observed recovery dynamics.

The fitted switching timepoint between response and recovery differed for ON- and OFF-cells (Figure 2g): $T_{\text{switch}}^{ON}=0.176\;s$ before withdrawal (97% HDI: 0.249–0.102 s before), and $T_{\text{switch}}^{\text{OFF}}=2.234\;s$ after withdrawal (97% HDI: 1.527–3.004 s after). Thus ON-cells peak slightly before withdrawal, while OFF-cells enter a brief post-withdrawal depression before beginning their recovery phase. This relative timing is visible in previous literature but has not been explicitly modeled^15^.

The response phase was modeled as an exponential rise for ON-cells and a sigmoidal decrease for OFF-cells. The recovery phase was parameterized using alternative functional forms: single vs. double exponential recovery for ON-cells, and double exponential vs. linear + exponential recovery for OFF-cells (Figure 2b).

As shown in Figure 2c,h, ON-cell recovery was best captured by a double exponential model, indicating two distinct timescales. ON-cell firing rose on a sub-second timescale before paw withdrawal, with time constant $\tau_{\text{pre}}=\;0.481\;s$ (97% HDI: 0.401–0.564 s). After paw withdrawal, the recovery exhibited both a fast component $\tau_{\text{fast}}=\;2.850\;s$ (97% HDI: 1.735–3.913 s) and a slow component $\tau_{\text{slow}}=\;61.252\;s$ (97% HDI: 47.781–75.855 s). A single exponential model (Figure 2e) could not simultaneously capture the sharp evoked peak and the prolonged decay, instead over- and undershooting different portions of the trajectory.

For OFF-cells, recovery was best described by the combined linear and exponential model (Figure 2d,i). OFF-cell firing declined sharply before withdrawal, with a sigmoidal response slope $k=2.895{\;s}^{-1}$ (97% HDI: 2.280–3.608 s^−1^). The post-withdrawal recovery contained a fast exponential component τ=4.206s (97% HDI: 3.250–5.081 s), together with a slower linear drift of $k_{\text{linear}}=0.063$ counts per second (97% HDI: 0.055–0.072 counts per second). Although the double exponential alternative fit the early part of the recovery (Figure 2f), it failed to asymptote to baseline over the recorded interval, making the linear+exponential model the better descriptor of the OFF-cell dynamics we observed.

We next quantified the timing of the response and recovery phases of the evoked changes in firing rate in terms of percentage deviations from baseline (see Supplemental Figure 1 for full posteriors). OFF-cell population firing had already decreased by 10% from baseline at 1.23 s before the paw withdrawal (97% HDI: 1.48–0.97 s), and ON-cell firing increased by 10% on a similar timescale (97% HDI: 1.5–1.1 s), indicating that both populations depart from baseline well before the behavioral response. The two classes also crossed 50% of their total change within a narrow pre-withdrawal window (OFF: 454 ms before; 97% HDI: 578–336 ms; ON: 500 ms before; 97% HDI: 570–450 ms), showing that the dominant portion of the evoked response is temporally locked to the upcoming reflex. Notably, ON-cell firing reached 90% of its peak well before paw withdrawal (220 ms before, 97% HDI: 320–160 ms), while OFF-cell suppression did not reach 90% until after the behavioral response (313 ms post, 97% HDI: 93–528 ms), reflecting a temporal asymmetry in how the two populations complete their evoked transitions.

Comparison of these values to the transition time for OFF-cells described above shows that OFF-cell firing continues to decrease for several seconds post-withdrawal before beginning to recover. At the population level, ON-cell activation both precedes withdrawal and completes its evoked component rapidly, whereas OFF-cell suppression is slower to reach its minimum and persists into the post-withdrawal period. Consistent with this ordering, ON-cell activation reaches its peak before OFF-cell firing reaches full suppression, indicating that the shift toward an ON-dominant state begins before the OFF-dominant state has fully terminated. Because this timing was observed in a pseudo-population rather than within simultaneously recorded ON/OFF pairs, it should be interpreted as a population-level temporal organization rather than a claim about pairwise interactions.

We used separate baselines for the pre- and post-withdrawal activity, which allowed us to quantify recovery on the same percentage scale, accounting for any slow modulation of tonic firing relative to the trial length. ON-cells returned to 50% of their post-withdrawal baseline by 10.84 s after the withdrawal (97% HDI: 6.175–16.34 s), but required more than an order of magnitude longer to approach full recovery, reaching 90% of their baseline by 107.7 s (97% HDI: 80.68–139.4 s). OFF-cells increased back to 50% of their baseline by 8.4 s post-withdrawal (97% HDI: 7–9.8 s) but to 90% of their baseline by 74 s (97% HDI: 70–78 s). These timescales are considerably longer than the typical duration of EMG activity^15^, indicating that the prolonged temporal structure cannot be explained as a trivial consequence of the motor output itself.

An important consequence of modeling ON- and OFF-cell activity parametrically is that it allows the sharpness of the evoked transitions to be quantified directly, using the slope or time constant of the transitions, rather than inferred qualitatively from peri-stimulus averages (as in Figure 1c)^18^. For OFF-cells, the slope of the sigmoidal fits to firing-rate decreases was significantly steeper for the withdrawal-aligned data (2.895 total spikes per bin, 97% HDI: 2.280–3.608 spikes per bin), compared to the heat-aligned data (1.858 total spikes per bin, 97% HDI: 1.514–2.235 spikes per bin) (Supplementary Tables 7 and 8). Similarly, ON-cells exhibited faster increases in firing when aligned to withdrawal, with exponential time constants of 0.481 s (97% HDI: 0.401–0.564 s), compared to 0.949 s (97% HDI: 0.812–1.083 s) when aligned to heat onset (Supplementary Tables 4 and 5). In contrast, NEUTRAL-cells (*n* = 32; 69 withdrawal-aligned and 70 heat-aligned trials) exhibited no significant modulation associated with either heat onset or withdrawal behavior (Supplementary Figure 3).

Taken together, these results shed new light on the traditional reflex-locked view and demonstrate that ON- and OFF-cell dynamics unfold on multiple, cell-type–specific timescales that extend well beyond paw withdrawal. This led us to examine whether ON- and OFF-cells exhibit ongoing rhythmic activity in the absence of stimulation, and how such slow oscillations might interact with stimulus-evoked responses.

### RVM ON- and OFF-cells exhibit slow rhythmic structure during ongoing activity

**Figure 3:**
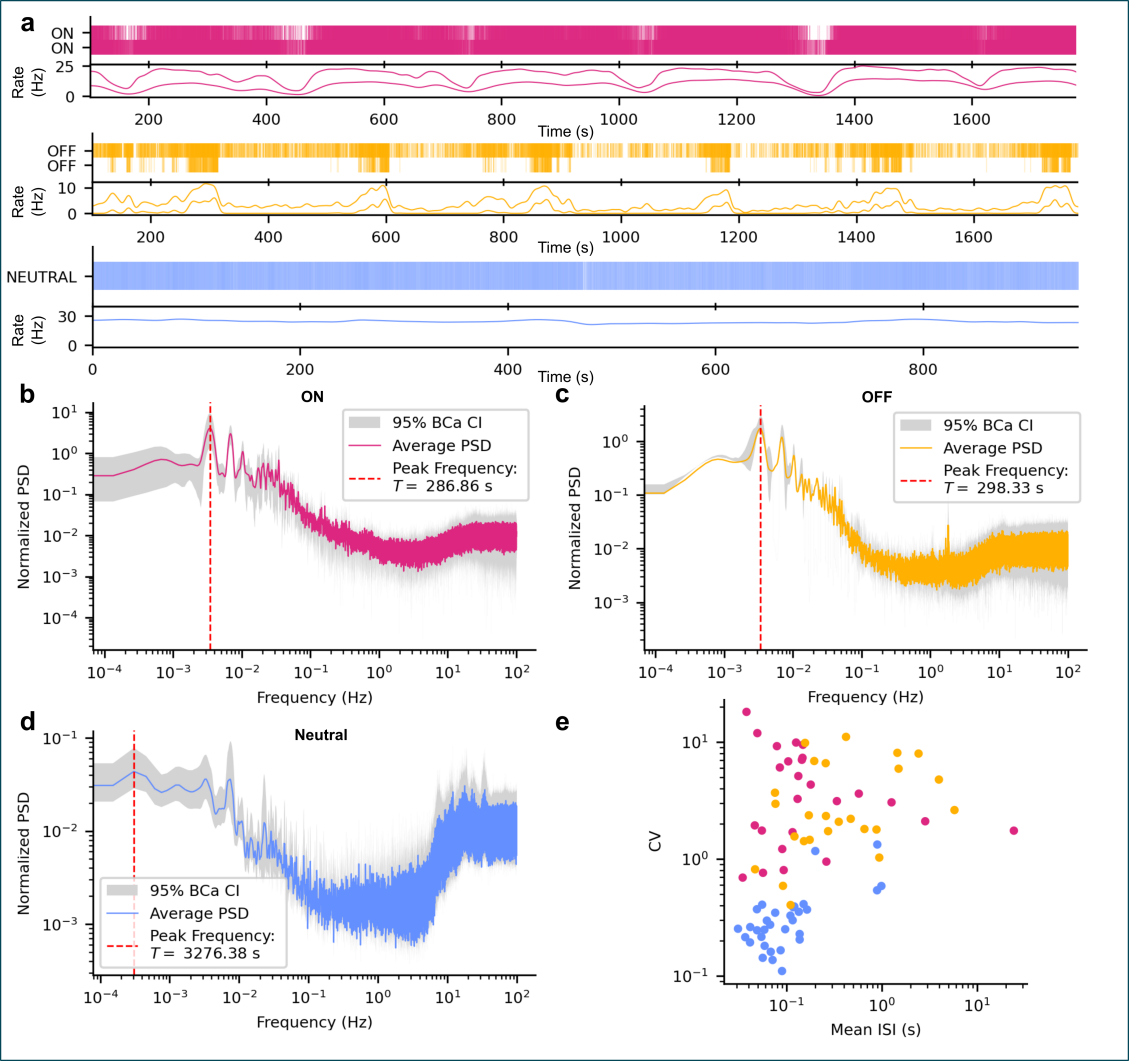
Ongoing activity of RVM neurons reveals distinct rhythmic signatures in ON- and OFF-cells. **a**, Example spike rasters (top) and rate plots (bottom) from simultaneously recorded ON-, OFF-, and NEUTRAL-cells during unstimulated periods. ON- and OFF-cells exhibit irregular, burst-like activity with slow co-fluctuations, whereas NEUTRAL-cells fire regularly at a high rate. **b**-**d**, Population-averaged power spectra of ongoing activity for ON-, OFF-, and NEUTRAL-cells. ON- and OFF-cells show clear low-frequency peaks just below *T* = 300 s (5 min), with associated harmonics, while NEUTRAL-cells lack such structure and instead show strong high-frequency components (10–100 Hz). **e**, Coefficient of variation (CV) versus mean interspike interval (ISI) for all recorded cells. NEUTRAL-cells form a distinct cluster characterized by low CV and short ISIs (regular high-rate firing), whereas ON- and OFF-cells show broad CV distributions and longer ISIs, consistent with irregular burst dynamics.

To investigate the ongoing activity of RVM neurons, we analyzed ON-, OFF-, and NEUTRAL-cells ($N_{\text{ON}\;}=25,N_{\text{OFF}\;}=25,N_{\text{NEUTRAL}\;}=32$) recorded in the absence of external stimulation (Figure 3a).

Even without sensory input, ON- and OFF-cells exhibited slow, rhythmic fluctuations in firing rates (examples in Figure 3a). To quantify these apparent rhythms, we computed power spectral density (PSD) estimates for each cell class (Figure 3b,c,d). Both ON- and OFF-cells exhibited clear low-frequency spectral peaks, often accompanied by harmonics, indicating quasi-periodic dynamics with a characteristic period of approximately five minutes ( $T_{\text{peak}\;}^{ON}=286.86\;s$, full width at half maximum (FWHM) = 16.92 s; $T_{\text{peak}}^{OFF}=\;298.33\;s$, FWHM = 16.12 s). NEUTRAL-cells, in contrast, lacked consistent spectral peaks, instead showing broad low-frequency components and prominent high-frequency power (> 10 Hz), reflecting regular, tonic firing. These results reveal a slow rhythmic modulation seemingly specific to ON- and OFF-cells.

We next summarized firing variability over the full recording period (Figure 3e). NEUTRAL-cells fired regularly and rapidly (Coefficient of Variation = 0.35 ± 0.26; mean InterSpike Interval = 0.17 ± 0.25 s, ~ 10 Hz). In contrast, ON- and OFF-cells exhibited highly variable, burst-like firing patterns, with coefficients of variation well above 1 (${CV}_{\text{ON}}=\;4.91\;\pm \;4.32,\;{CV}_{OFF}\;=\;3.70\;\pm \;3.09$) and broad interspike interval (ISI) distributions (${ISI}_{ON}=1.25\;\pm \;4.80\;s,\;{ISI}_{OFF}=0.84\;\pm 1.36\;s$; Supplementary Table 2). Because relatively few neurons were recorded per animal, we did not attempt further subclustering.

Together, these analyses indicate that ON- and OFF-cells exhibit structured, rhythmic fluctuations in ongoing activity, whereas NEUTRAL-cells maintain high-rate, non-rhythmic firing. However, frequency-domain measures such as PSD and summary statistics capture only average power and variability; they discard temporal phase information and cannot resolve how rhythmic firing unfolds over time.

To address this, we next used Gaussian process (GP) modeling to characterize ongoing dynamics directly in the time domain. We implemented two complementary GP approaches: a purely periodic GP with a Gaussian likelihood to identify dominant rhythmic timescales, and a generalized Poisson GP with a SoftPlus link function to test whether these slow fluctuations were structured and predictable on a per-neuron basis.

### A periodic Gaussian process reveals dominant slow oscillations in ON- and OFF-cell activity.

**Figure 4:**
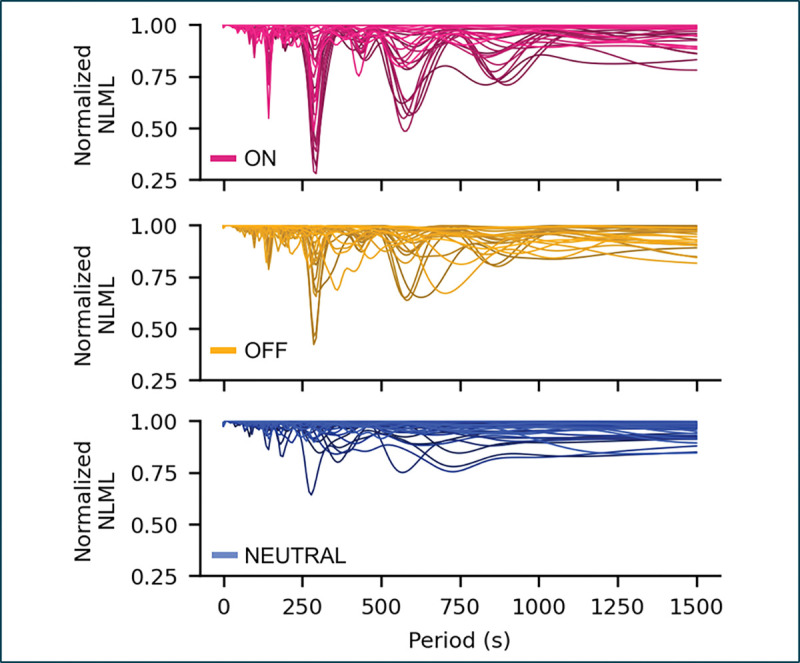
Period-length scans of the negative log marginal likelihood (NLML) from periodic Gaussian process fits to ongoing firing in ON-, OFF-, and NEUTRAL-cells. ON- and OFF-cells show distinct minima at ~ 300 s and its harmonics, whereas NEUTRAL-cells exhibit flat profiles.

To identify the characteristic timescales underlying the slow fluctuations observed during ongoing activity, we applied a periodic GP model with a Gaussian likelihood to the smoothed firing rates of each neuron, scanning across 200 candidate periods (0.1–1500 s). This model quantifies how strongly each frequency explains the observed firing pattern, assuming only that the waveform is smooth and periodic (Figure 4). ON- and OFF-cells showed clear minima in the negative log marginal likelihood (NLML) near 300 s, with additional subharmonic peaks at 600 and 900 s, indicating quasi-periodic dynamics with a dominant 5-minute cycle. In contrast, NEUTRAL-cells (with one exception) displayed flat NLML profiles with no consistent minima, without evidence of stable rhythmicity. These results establish that slow oscillations are a robust and reproducible feature of ON- and OFF-cell activity.

### A Poisson Gaussian process predicts slow rhythmic fluctuations in ongoing RVM firing

**Figure 5:**
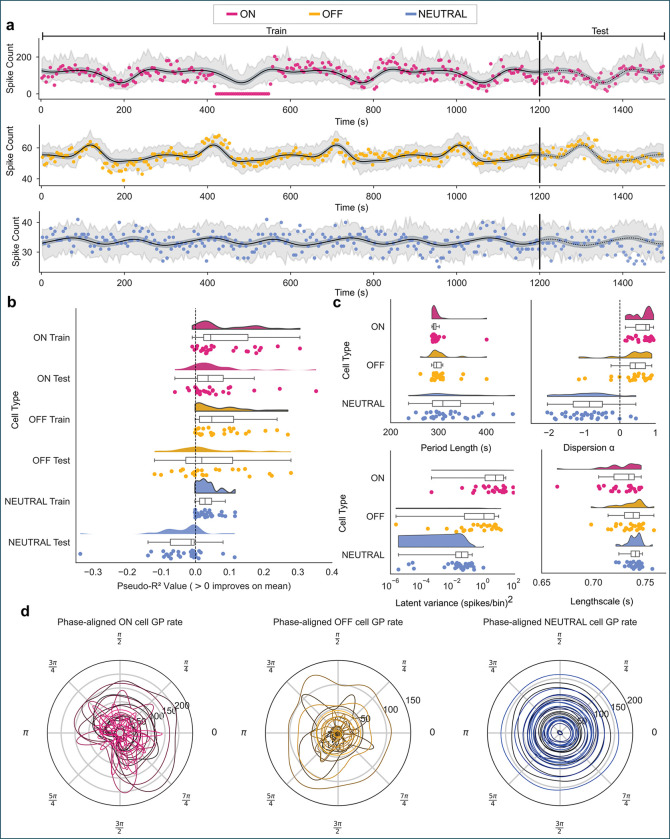
Periodic Gaussian process (GP) modeling of ongoing ON-, OFF-, and NEUTRAL-cell activity. **a**, Example fits for single ON-, OFF-, and NEUTRAL-cells using a periodic GP kernel. GP fits were robust to noise or occasional periods of silence in the training data (e.g. ON-cell trace, 410–550 s). Models were trained on all data except the final 300 s (training region: solid line) and tasked with predicting the held-out test segment (dotted line), without using test data during fitting. **b**, Pseudo-$R^{2}$ values for training and test sets. ON-cells achieve significant predictive performance on test data, OFF-cells show weak predictability, while NEUTRAL-cells fail to generalize (test Pseudo-$R^{2}<0$). **c**, Posterior hyperparameters across cell classes. ON- and OFF-cells exhibit positive dispersion parameters *α* and tightly clustered period estimates near 300 s, consistent with quasi-periodic firing. NEUTRAL-cells show negative dispersion and less coherent period estimates, reflecting their regular, high-rate activity. **d**, Phase aligned GP rates adjusted for found period length showed unique signatures for each cell.

We next asked whether these slow oscillations were predictable—that is, whether past firing reliably forecasted future activity. To test this, we used a Poisson GP model with a SoftPlus link function, allowing the variance of firing rates to scale with their mean through a dispersion parameter (*α*). Each model was trained on all but the final 300 s of data and tasked with predicting the held-out test segment (Figure 5a). The fitted models (solid lines) accurately captured slow fluctuations during training and generalized into the test window (dotted lines) robustly for ON-cells, variably for OFF-cells, and not for NEUTRAL-cells.

Training and test accuracies, quantified by pseudo-$R^{2}$, were computed for each cell (Figure 5b). We performed two-sided T-tests to assess significance ($R^{2}\;\neq \;0$) using sign flipping permutations of the $R^{2}$ value of all cells per animal, and the median $R^{2}$ at each permutation (see Supplemental Figure 4 for distributions). This accounted for the potential correlation of ON- and OFF-cells recorded from the same animal. All three cell classes achieved significant training accuracy ($p_{ON}=0.0047,p_{OFF}=0.0004,p_{\text{NEUTRAL}\;}<0.0001$). Comparing $R^{2}$ values using a similarly permuted and Bonferroni corrected Mann Whitney U test revealed no significant differences between training $R^{2}$ for the three groups (see Supplemental Figure 6). This was expected, since even apparently aperiodic firing rates can contain some variance which can be captured by the GP. Therefore, we turned to the predictive accuracy to confirm the periodicity of each cell class, based on the predictions given by the periodic kernel.

By contrast, test predictability, quantified by pseudo-$R^{2}$, dissociated the three populations (Figure 5b). ON-cells showed significant generalization to unseen data (p=0.0067), with the large majority of cells exceeding baseline performance (i.e., >0). OFF-cells, as a population, did not show reliable predictive structure (p=0.3127), reflecting a mixture of well-predicted and poorly predicted neurons. NEUTRAL-cells performed significantly below baseline (p=0.0033), demonstrating a lack of periodicity. Group comparisons revealed significant differences between NEUTRAL-cells and both ON- and OFF-cells (p<0.001 and p=0.006, respectively), while ON- and OFF-cells did not differ significantly at test (p=0.939). Kruskal–Wallis tests confirmed significant group differences at test time (p<0.001) but not during training (p=0.117). Thus, reproducible long-timescale predictability is a defining feature of ON-cells, while OFF-cells exhibit oscillatory structure that is present but less consistent across neurons.

Posterior hyperparameters (Figure 5c; Table 1) revealed further distinctions among cell classes. Estimated oscillation periods for both ON- and OFF-cells clustered near 300 s, corroborating the frequency-domain analyses. Although NEUTRAL-cells also displayed nominal period clustering near 300 s, their low latent variance and poor predictive performance indicate that this reflects weak parameter constraint rather than true rhythmic structure.

ON- and OFF-cells exhibited positive dispersion parameters $\left(\alpha >0;p_{ON}=0.0011,p_{OFF}=0.0105\right)$, consistent with bursty, overdispersed firing. In contrast, NEUTRAL-cells showed negative dispersion ($p_{\text{NEUTRAL}}<0.001$), indicating highly regular firing with variance below Poisson expectation. Latent GP variance further separated the classes: ON-cells showed the largest modulation amplitude, OFF-cells intermediate variance, and NEUTRAL-cells near-flat latent structure.

Finally, single-cycle phase-normalized reconstructions (Figure 5d) revealed cell-specific oscillatory signatures in ON- and OFF-cells, whereas NEUTRAL-cells showed near-constant phase profiles, consistent with an absence of structured rhythmic modulation.

Together, these results demonstrate that slow, quasi-periodic fluctuations in RVM activity are most consistently expressed and predictably organized in ON-cells, present but heterogeneous in OFF-cells, and absent in NEUTRAL-cells. This structure indicates that ongoing RVM dynamics are not stochastic but reflect organized network states operating on multi-minute timescales.

### Low-frequency coherence between RVM activity and heart rate

The RVM is implicated not only in nociceptive modulation but also in autonomic control, influencing cardiovascular and respiratory outputs^9–11,19–21^. Given this dual role, we next asked whether the slow RVM oscillations identified above might couple to an autonomic variable—specifically, heart rate, a key homeostatic parameter that itself exhibits rhythmic fluctuations.

Previous studies using small samples of ON- and OFF-cells reported correlations between ON/OFF-cell firing and cardiovascular measures such as heart rate and blood pressure^22,23^. Here, we extended these findings by testing whether slow RVM oscillations and heart rate exhibit a consistent phase relationship using coherence analysis, a frequency-domain measure that detects shared periodic structure even in the presence of noise or latency offsets.

**Figure 6:**
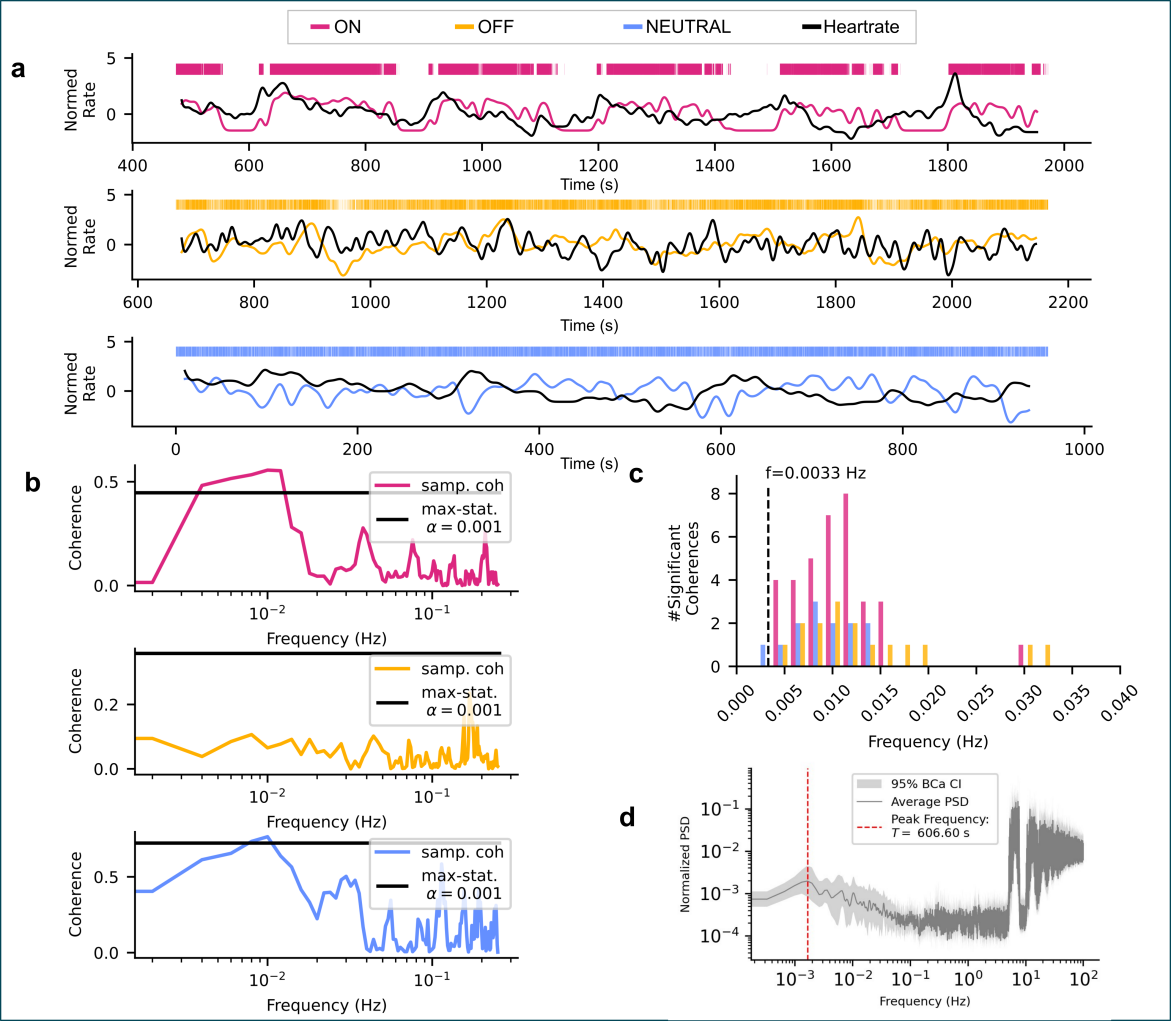
Analysis of heart rate coherence against cell firing rate. **a**, Example normed firing rates and heart rates for ON-, OFF-, and NEUTRAL-cells, showing both visibly coherent and incoherent activity. **b**, Coherence against frequency for the same cells in (**a**), showing the max-statistic significance level (black line). **c**, Total number of significant coherences per frequency for all cells. **d**, Combined averaged heart rate power spectrum over all animals, showing clear heartbeat-related peaks > 1 Hz as well as low frequency peaks. Lowest frequency peak indicated in red.

The neuronal firing rates and simultaneously recorded heart rates (Figure 6a) were segmented and demeaned, and coherence estimates were computed for each cell (Figure 6b). A subset of cells (5/32 NEUTRAL, 4/25 OFF, 8/25 ON) showed significant low-frequency coherence with heart rate (Figure 6c). Although the peak coherence frequencies did not exactly match the dominant ON- and OFF-cell oscillation period (~ 300 s), spectral peaks in heart rate were observed at multiples of this period (Figure 6d), suggesting that RVM activity and cardiac rhythms share a common underlying regulatory timescale.

These results provide preliminary evidence for partial coupling between descending pain-modulating activity and autonomic rhythms, consistent with the integrative role of the RVM in coordinating sensory and homeostatic processes.

## Discussion

In this study, we set out to characterize how cells of the rostral ventromedial medulla (RVM) operate across time—both during reflex-evoking stimulation and in the absence of external input. By combining single-unit recordings with probabilistic modeling, we show that ON- and OFF-cells, the principal modulators of spinal nociceptive processing, express multiple coordinated timescales of activity: rapid, stimulus-evoked responses unfolding over seconds to tens of seconds, and slow, quasi-periodic fluctuations in ongoing firing occurring over minutes. These findings reveal that descending pain-modulatory circuits are not purely reactive but instead exhibit a temporally organized structure that integrates reflexive and state-dependent processes—a perspective that fundamentally reframes how RVM function is understood.

Bayesian modeling of withdrawal-aligned activity revealed that ON- and OFF-cells exhibit structured, multi-phase responses rather than simple bursts or pauses. An advantage of our approach is that it jointly models the evoked response and recovery phases, providing posterior estimates of peak and nadir firing and dissociating these extrema from the withdrawal event itself. This allowed us to evaluate alternative temporal parameterizations and identify the specific combination of timescales needed to explain the population trajectories. As expected, the resulting fits captured the canonical withdrawal-related changes in firing described in classical RVM work^5^ and quantitatively demonstrated that population activity is more tightly aligned to the behavioral withdrawal than to the onset of noxious stimulation.

The same analysis uncovered a previously unrecognized population-level temporal relationship between the two cell classes. Although paired ON/OFF-cell recordings show that the first spike of the ON-cell burst follows the onset of the OFF-cell pause, our pseudo-population modeling revealed that the peak of ON-cell activity precedes both the withdrawal and the nadir of OFF-cell suppression. ON-cells peaked 180 ms before withdrawal, whereas OFF-cells reached their minimum more than 2 s after it. Thus, although the two classes are anti-correlated on fast timescales, their extrema are not simply mirrored. Instead, facilitation and inhibition are temporally offset in a structured manner, with ON-cell activity leading the reflex and OFF-cell suppression persisting well beyond it.

Analysis of the recovery phase revealed multiple timescales in both populations. ON-cell recovery was best captured by a double-exponential function, whereas OFF-cell recovery was best described by a combined linear and exponential trajectory. Neither population returned to its pre-stimulus firing rate within 100 s. This separation of fast and slow components suggests distinct biological processes underlying the initial response and the prolonged return toward baseline. The abrupt OFF-cell pause and sharp ON-cell peak are consistent with positive-feedback mechanisms that support rapid, precisely timed actions, whereas the slower recovery component aligns with the prolonged adjustments in nociceptive responsiveness observed behaviorally. Consistent with this, blocking the OFF-cell pause suppresses withdrawal^24^, and attenuating the ON-cell burst reduces reflex magnitude^25,26^. Moreover, the slow post-withdrawal drift toward baseline correlates with increased sensitivity to subsequent stimuli^27^ and with altered ON/OFF-cell balance in persistent pain models (e.g., spinal nerve ligation, CFA inflammation^28,29^). Together, these findings suggest that the initial response and its hysteresis-like continuation serve complementary roles: coordinating the reflex itself while shaping a longer-lasting nociceptive state in the face of potential threat.

More broadly, while temporal hierarchies linking fast sensory responses to slower, state-dependent fluctuations are well established in cortical and neuromodulatory systems^30,31^, our results show that a similarly organized architecture operates within descending pain modulatory pathways—a level of the nociceptive system where such temporal structure had not previously been quantified. This places the RVM within a distributed temporal hierarchy in which rapid reflex-related signals and slow internal-state dynamics jointly structure behavior.

In this framework, ON- and OFF-cells are not simple reciprocal antagonists. Rather, they contribute distinct and temporally staggered components of descending modulation—consistent with their different pharmacologies and the fact that each population can be manipulated independently. The multi-timescale organization revealed here offers a mechanistic basis for understanding how RVM populations support both rapid nocifensive reactions and slower adjustments in nociceptive tone.

Beyond event-related responses, we identified slow, quasi-periodic oscillations in ongoing firing, with a dominant period near five minutes. These oscillations, evident in both the power spectrum and Gaussian process models, were consistent across animals and cell classes but not evident in NEUTRAL-cells, suggesting that they reflect an intrinsic property of the pain-modulatory network rather than a global effect (e.g., anesthesia). The presence of predictable low-frequency structure implies that the RVM maintains a temporally organized baseline, alternating between facilitative (ON-dominant) and inhibitory (OFF-dominant) modes even in the absence of noxious input. Although we did not observe phase-dependent effects on withdrawal latency (Supplemental Figure 10), the experiment was not designed to test this directly. Prior work demonstrating increased nociceptive sensitivity during ON-cell dominance^3,32^ suggests that such internal dynamics could still bias behavioral responsiveness under natural conditions.

The origin of these slow oscillations remains unresolved. Given the limited local connectivity among ON- and OFF-cells^33^, the rhythmicity is unlikely to arise from intrinsic microcircuit loops and more plausibly reflects intrinsic membrane properties of these cells or broader feedback interactions involving the spinal cord, periaqueductal gray, hypothalamus, or other brainstem nuclei^3,6,34–36^. The strong synchrony within each functional population, combined with pronounced asymmetries between them, suggests coordination via shared modulatory inputs rather than reciprocal microcircuitry.

This circuit architecture intersects with another key feature of the RVM: substantial molecular heterogeneity within ON- and OFF-cell populations^37–39^. Despite preventing a straightforward molecular classification of ON- and OFF-cells, such diversity may support the flexible functional organization observed here, enabling the integration of upstream control signals based on both internal (slow periodicity) and external (stimulus-evoked) variables. The RVM receives convergent non-nociceptive inputs^40^ and participates in thermoregulatory and autonomic processes^23,41^, further supporting a role as a multimodal hub integrating information across modalities and timescales.

Pain modulation is closely intertwined with autonomic and behavioral adjustments that support homeostasis^7,42,43^. Prior work reports associations between ON/OFF-cell firing and heart rate or body temperature^22,23^. Motivated by this, we examined heart rate as a potential covarying signal. Because both heart rate and RVM activity are shaped by many independent factors, simple correlation is difficult to interpret; coherence provides a more targeted measure of shared structure at the timescale of interest. A subset of neurons across all three cell classes showed significant low-frequency coherence with heart rate, and heart-rate spectra exhibited peaks at the RVM oscillation frequency and its harmonics. We interpret these findings cautiously: they indicate that the oscillation is detectable outside the RVM and therefore physiologically meaningful but do not imply direct causation. Notably, coherence in some NEUTRAL-cells—despite their lack of intrinsic rhythmicity—suggests that modulatory inputs influencing the slow oscillation distribute across RVM cell types and underscore the still-unclear functional role of NEUTRAL-cells.

In conclusion, these results demonstrate that the RVM operates across multiple concurrent timescales, from seconds-long evoked responses to minute-scale endogenous rhythms, providing quantitative evidence that descending pain modulation is temporally organized rather than continuously graded. This temporally layered architecture offers a framework for understanding how sensory processing is coordinated with internal state and highlights the importance of dynamics as a dimension of brainstem function. Future work, especially multichannel recordings and causal manipulations in awake animals, will be essential for determining how these temporal processes shape behavior and broader physiological regulation.

## Methods

### Experiments

All experiments followed the guidelines of the National Institutes of Health and the Committee for Research and Ethical Issues of the International Association for the Study of Pain, and were approved by the Institutional Animal Care and Use Committee at the Oregon Health & Science University (OHSU).

Male and female (n = 78 and 5 respectively) Sprague-Dawley rats (Charles River; 191–348 g, mean ± SD: 264 ± 27 g) were deeply anesthetized using isoflurane (4–5%) and a catheter placed in the external jugular vein for subsequent infusion of the short-acting barbiturate methohexital. They were then transferred to a stereotactic frame and a small craniotomy made to gain access to the RVM. Body temperature was monitored and maintained at 36–37 °C with a heating pad, and heart rate was monitored using EKG. Mean ± SD heart rate was 391.1 ± 34.4 bpm, with mean range over the protocol 0.62 ± 12.0 bpm across all animals.

After preparatory surgery was complete, the anesthetic plane was lowered such that a heat stimulus applied at 5-min intervals to the plantar surface of the hindpaw (radiant or contact heat delivered using a Peltier device) elicited a paw withdrawal. The stimulus consisted of a ramp from 36 to 53 °C over a period of 11 s. The stimulus was terminated when the animal withdrew the paw (mean ± SD paw withdrawal temperature: 49.0 ± 2.0 °C, latency: 8.7 ± 1.1 s). The paw surface was maintained at 35 °C between trials. Recordings were performed between August 2020 and August 2024.

Once the animal responded to the heat stimulus, methohexital was infused at a rate (37.8–77.5 mg/kg/hr, mean ± SD: 60.0 ± 7.6 mg/kg/hr) that maintained a stable anesthetic plane (as indicated by stable paw withdrawal response) while preventing spontaneous movement^44,45^. The experiment was performed in low ambient light conditions (< 10 lux). Extracellular single-unit recordings were acquired using a stainless-steel microelectrode (Frederick Haer & Co). Signals were amplified (10k) and band-pass filtered (400 Hz to 15 kHz, Neurolog, Digitimer) before analog-to-digital conversion at 32k samples/s for real-time spike detection and monitoring using Spike2 software (CED, Cambridge, UK). Correct waveform identification was verified on an individual spike basis at the conclusion of the experiment using Spike2 template matching and cluster analysis. EMG activity (to monitor withdrawal reflexes) was also recorded using Spike2. An RVM neuron or neurons was isolated and characterized as an ON-, OFF-, or NEUTRAL-cell based on changes in firing rate associated with withdrawal of the paw from a noxious mechanical stimulus applied to the hindpaw^13,46^. ON-cells are defined by a period of activity beginning just prior to withdrawal from a noxious stimulus (or maintained firing if already active at the time of stimulus delivery). OFF-cells stop firing just prior to withdrawal (or remain silent if inactive). NEUTRAL-cells do not respond. We performed/analyzed data from two complementary protocols, each designed to capture different aspects of RVM activity during noxious stimulation or extended unstimulated periods. In the “evoked” protocol, noxious heat stimuli were delivered at 5 min intervals over a period of 16–18 min. A total of 104 cells were recorded in 53 animals: 35 OFF-cells, 45 ON-cells, and 24 NEUTRAL-cells. The “ongoing” protocol was designed specifically to examine ongoing activity in the absence of noxious stimulation. Cell activity was monitored for a period of 30 min following an initial noxious heat trial to classify the cell under study. Evoked responses were confirmed in at least two trials at the end of the experiment. Using this protocol, we recorded 62 cells in 30 animals: 25 OFF-cells, 25 ON-cells, and 12 NEUTRAL-cells. Together these protocols provided both noxious-evoked and prolonged recordings of ongoing, or “spontaneous” activity of ON-, OFF-, and NEUTRAL-cells, enabling systematic analysis of stimulus-driven dynamics and slow ongoing fluctuations. A detailed breakdown of cell numbers and animals used in each analysis is provided in Supplemental Table 10.

Recording sites in RVM were marked with an electrolytic lesion at the conclusion of the experiment. Animals were overdosed with methohexital, and perfused transcardially with saline followed by 10% formalin. Brains were removed and post-fixed for 24 h in 10% formalin, then equilibrated for 24–72 h in 30% sucrose in PBS at 4 °C. Brains were sectioned at 40 to 60 μm, and recording sites were plotted (Supplementary Figure 11). The RVM was defined as the nucleus raphe magnus and adjacent reticular formation medial to the lateral boundary of the pyramids at the level of the facial nucleus.

### Data Processing

After spike sorting and manual classification of cells, a window around each trial (−10 s to 100 s) was extracted relative to the alignment marker (heat onset or withdrawal behavior). Spikes within each window were aggregated across animals and trials to form the PSTHs for ON- and OFF-cells, providing a population-level view of stimulus-evoked activity. Trials were excluded from PSTH construction if the recording ended within the window or if the next event (heat onset or withdrawal) truncated the usable interval, resulting in a variable number of trials per PSTH.

For cells with available ongoing-activity recordings, we extracted a 1500 s segment (or 960 s for the shorter NEUTRAL-cell datasets) relative to the start of the first post–ongoing-activity trial. This ensured a minimum 230 s buffer after the initial trial, allowing transient evoked activity to dissipate before analyses of ongoing dynamics. Because noxious stimulation had no effect on NEUTRAL-cell firing rates, the full recording period from all neutral cells was used for GP modeling.

All analyses were conducted in Python. Instantaneous firing rates and basic statistics were computed using the Elephant package^47^. Gaussian processes were implemented using GPyTorch and GPy^48,49^. Code interfaced with the Neo framework and the SonPy package to convert Spike2 (.smrx) files into Neo objects^50,51^. PyMC was used for Bayesian regression^52^ .

We used Bayesian regression to model stimulation-evoked responses because the models contain relatively few parameters and the Bayesian framework provides full posterior uncertainty estimates—particularly useful for the switching timepoint. For ongoing activity, we employed maximum-likelihood type-II GP regression with a generalized Poisson likelihood. This approach was required because the long recording durations (> 1500 s), the nonstandard likelihood, and the multiple local minima induced by the periodic kernel made full Bayesian sampling computationally prohibitive.

### Piecewise Bayesian Modeling of ON- and OFF-cells

We modeled the activity of a pseudo-population composed of cells and trials from multiple animals, rather than modeling each cell or trial individually in a multi-level model. The reasons for this were twofold: firstly, with only 3–4 trials per neuron, and roughly 2 neurons per animal, there was not enough data to estimate a full multi-level model. Secondly, as mentioned above, our primary aim was to capture population-level structure. Single-electrode recordings are essentially random samples from the population of ON- and OFF-cells, and we therefore assumed the emergence of population level effects to be due to exchangeability and uncorrelated noise averaging out across neurons.

We used piecewise Bayesian regression to fit the dynamics of ON- and OFF-cells during trials with noxious stimulation. We binned spikes using a 50 ms time bin, assuming that the total spike count for ON- or OFF-cells per bin $y_{t}^{ON}$ was Poisson distributed with a variable rate $\lambda_{t}$, dependent on parameters θ, such that:

(1) $$y_{t}^{ON}∼Po\left(\lambda_{t}^{ON}\right)$$

(2) $$\lambda_{t}^{ON}=\left\{\begin{array}{ll} f_{\text{response}}(\theta ,t), & t<t_{\text{switch}}\\ f_{\text{recovery}}(\theta ,t), & t\geq t_{\text{switch}\;} \end{array}\right.$$

For ON-cells, the response phase was modeled as an exponential rise^18^. We compared single- and double-timescale recovery functions. The double exponential model consisted of:

$$\lambda_{t}^{ON}=\left\{\begin{array}{ll} r_{\text{pre}\;}+k_{\text{pre}\;}e^{\left(t-t_{s}\right)/\tau_{1}} & \;\text{if}\;t\leq t_{s}\\ r_{\text{post}\;}+k_{\text{fast}\;}e^{\left(t-t_{s}\right)/\tau_{2}}+k_{\text{slow}\;}e^{\left(t-t_{s}\right)/\tau_{3}} & \;\text{if}\;t>t_{s} \end{array}\right.$$

with continuity enforced by a corrected constraint:

$$k_{\text{slow}\;}=k_{\text{pre}\;}+r_{\text{pre}\;}-r_{\text{post}\;}-k_{\text{fast}\;}$$

Priors were:

$$log\;r_{\text{pre}\;},log\;r_{\text{post}\;}∼𝒩\left(\mu_{\text{pre}\;}=3.0,\sigma_{\text{pre}\;}=1.0\right)$$

$$log\;k_{\text{pre}\;},log\;k_{\text{post}\;}\;∼𝒩\left(\mu_{k}=1.0,\sigma_{k}=1.0\right)$$

$$\beta_{1},\beta_{2},\beta_{3}∼HalfNormal(\sigma =5.0)$$

$$t_{s}∼U[-4,4]$$

$\text{with}\;\tau_{i}=1/\beta_{i}$

The single exponential model for ON-cells was identical except for removal of the $k_{\text{fast}}$ term.

For OFF-cells we used a sigmoidal function as the response function^18^ and compared a double timescale recovery with an exponential + linear recovery function. We constrained the sigmoid to pass through the point ($t_{s},u_{0}$), joining the left and right side of the model. The general sigmoid has four parameters, slope k, midpoint $x_{0}$, lower asymptote a and upper asymptote b, such that:

$$f(t)=b+\frac{a-b}{1+e^{-k\left(t-t^{*}\right)}}$$

We fitted a,b and k directly; however, using a reparametrization of the sigmoid, we chose $t^{*}$ so that $f\left(t_{s}\right)=u_{0}$. Starting from the relation:

$$u_{0}=b+\frac{a-b}{1+e^{-k\left(t_{s}-t^{*}\right)}}$$

we then solve for $x_{0}$, obtaining finally:

$$t^{*}=t_{s}+\frac{1}{k}ln\left(\frac{a\;-\;b}{u_{0}\;-\;b}-1\right)$$

The full model consisted of:

$$\lambda_{t}^{OFF}=\left\{\begin{array}{ll} b+\frac{a-b}{1+exp\left(-k\left(t-t^{*}\right)\right)} & \;\text{if}\;t\leq t_{s}\\ u_{0}+\left(c-u_{0}\right)\left(1-e^{-\left(t-t_{s}\right)/\tau }\right)+k_{3}\left(t-t_{s}\right) & \;\text{if}\;t>t_{s} \end{array}\right.$$

For the double exponential case, the right hand side of the model ($t>t_{s}$) was replaced with:

$$u_{0}+\left(c-u_{0}\right)\left(1-e^{-\left(t-t_{s}\right)/\tau_{\text{slow}\;}}\right)+\left(d-u_{0}\right)\left(1-e^{-\left(t-t_{s}\right)/\tau_{\text{fast}}}\right)$$

Priors for the sigmoid were set as:

b∼HalfNormal(σ=4.0)

a,c∼b+HalfNormal(σ=4.0)

$$k,k_{2}∼𝒩(\mu =0.0,\sigma =1.0)$$

$$k_{3}∼HalfNormal(\sigma =1.0)$$

$$u_{0}∼U[a,b]$$

$$t_{s}∼U[-4.0,4.0]$$

and additional priors for the double exponential OFF-cell recovery d and $k_{3}$ were defined identically to c and $k_{2}$ for the linear + exponential case, respectively.

This Bayesian framework provided several advantages over prior methods^18^: (i) it does not require predefining “burst” and “pause” times; (ii) it dissociates peak and nadir timing from withdrawal; (iii) it yields full posterior distributions for extrema and firing rates at any time; and (iv) it enables principled comparison of alternative timescale parameterizations for recovery dynamics.

### Gaussian Processes modeling

Firstly, as a preliminary period scan across potential timescales of ongoing firing, a GP with a periodic covariance function (see below) and Gaussian likelihood was applied to the smoothed firing rates of each neuron. We used 200 candidate periods (0.1–500 s) and a fixed lengthscale and variance of 1.0 and 5.0, respectively. A smoothing kernel and sampling period, both of width 2 seconds, were used to transform the spikes to a rate. The first and last 2 seconds of data were removed to avoid artifacts due to the smoothing process.

Secondly, slow structure in ongoing firing was quantified using latent GP models applied to binned spike counts (5 s bins). For each neuron, we fitted a sparse variational GP (200 inducing points) with a constant mean and a periodic covariance function:

(3) $$x\left(t\right)∼𝒢𝒫\left(c,k\left(t,t^{\prime }\right)\right),$$

(4) $$k\left(t,t^{\prime }\right)=\sigma^{2}exp\left[-\frac{2{sin}^{2}\left(\pi \left|t-t^{\prime }\right|/p\right)}{\ell_{per}^{2}}\right],$$

where p denotes the oscillation period and $\ell_{\text{per}}$ the periodic length scale. A narrow Gaussian prior $\ell_{\text{per}}∼𝒩(0.1,0.1)$ was used to avoid over-smoothing in low-rate neurons. Kernel periods were initialized at 300 s, based on the period-scan analysis.

To map latent GP values to positive firing rates, a scaled SoftPlus transform,

(5) $$\mu_{t}=A\;log\;\left(1+exp\left(x_{t}\right)\right)$$

with A=10 spikes/s was used instead of the exponential link to prevent numerical instabilities and speed up convergence across both low and high-rate regimes.

Spike count variability exhibited substantial over- and under-dispersion across cell classes. We evaluated a range of likelihoods (Poisson, Conway–Maxwell–Poisson), kernels (periodic, spectral mixture), and link functions. A generalized Poisson model^53,54^ consistently provided stable fits and accommodated the broad dispersion range of ON-, OFF-, and NEUTRAL-cells. The generalized Poisson likelihood was parameterized by a parameter $\theta_{t}$ and dispersion parameter α:

(6) $$P\left(Y_{t}=y_{t}|\theta_{t},\alpha \right)=\frac{\theta_{t}{\left(\theta_{t}+\alpha y_{t}\right)}^{y_{t}-1}}{y_{t}!}\exp\left[-\left(\theta_{t}+\alpha y_{t}\right)\right].$$

$\theta_{t}$ was related to the mean $\mu_{t}$ by:

(7) $$\mu_{t}=E[Y]=\frac{\theta_{t}}{1-\alpha },\;|\alpha |<1.$$

We placed a prior α~𝒩(0,0.5) and computed $\theta_{t}=\mu_{t}(1-\alpha )$ at each time point. A small contamination term ($p_{\text{noise}}=0.001$) was added to account for rare outlier bins:

(8) $$p\left(y_{t}|\mu_{t}\right)=\left(1-p_{\text{noise}}\right)GPois\left(y_{t};\mu_{t},\alpha \right)\;\;+p_{\text{noise}}U\left[0,y_{max}\right].$$

### Coherence Estimation

We first binned each spike train x and heart rate y using a 2 s bin size. We split the recording into 500 s segments with a 50% overlap, which gave each segment frequency resolution 1/T=0.002Hz. We demeaned each segment then used a multitaper spectral estimator with discrete prolate spheroidal sequences (DPSS) (standardized half bandwidth NW = 3.5, number of tapers K=6) to calculate the coherence^55^, using the formula:

$$C_{xy}(f)=\frac{{\left|{\overset{‾}{S}}_{xy}(f)\right|}^{2}}{{\overset{‾}{S}}_{xx}(f){\overset{‾}{S}}_{yy}(f)}$$

Segments were then individually phase-randomized to build a surrogate coherence distribution, and used a max-statistic approach to control the Family-Wise Error Rate (FWER) across all frequencies. The significance threshold was set at α=0.001.

This combination of parameters gave a bandwidth = *B* = NW/*T* = 0.007 Hz, meaning that the frequency f=0.0033Hz(T=5 minutes) found by the Gaussian Process was smeared across the low-frequency end of our coherence estimator. It was therefore not possible to determine whether the significant coherences found were due to oscillations at the same frequency as the GP period, due to the constraints of recording length and robust spectral estimation. Between individual cells, we observed some visual and spectral differences across all cell types between 0.01 Hz “fast” coherence and 0.0033 Hz “slow” coherence but these could not be quantified.

## Supplementary Material

Supplement 1

## Figures and Tables

**Table 1:** Posterior hyperparameters from the fitted latent GP model.

Cell class	period *p* (s)	dispersion *α*	outputscale *σ*^2^	lengthscale *l*

ON	297 ± 22	0.63 ± 0.25	17 ± 26	0.73 ± 0.018
OFF	304 ± 27	0.36 ± 0.53	3.1 ± 4	0.74 ± 0.013
NEUT.	322 ± 54	−0.9 ± 0.63	0.1 ± 0.2	0.74 ± 0.0068

## Data Availability

Data and code will be openly available upon acceptance.

## 0.1 Code availability

Code will be made available on Zenodo code repository.
