# Supplementary material for: Multi-timescale Rhythmic Dynamics in Rostral Ventromedial Medulla Neurons
Source: bioRxiv. 2025 Dec 17:2025.12.15.693905. Preprint. [Version 1] doi: 10.64898/2025.12.15.693905 (PMC12724712; doi:10.64898/2025.12.15.693905)
Supplement: Supplement 1 [file media-1.pdf]

2 Supplementary Information

Spike Train Power Spectra

A spike train can be represented by a sum of delta functions $s(t) = \sum_{i=0}^{i=N} \delta(t - t_i)$. Therefore, the spectrum for a spike train can be directly computed as a sum over complex exponentials at each spike location, which can be evaluated at any frequency. However, due to the number of spikes and the computational benefits of using the FFT algorithm to compute the PSD, it is conventional to first bin the spikes with a fine bin width (e.g. 5 ms). The resulting sequence, which tends to a binary sequence as the bin width becomes small relative to the average spacing between spikes, is then used to compute the PSD using conventional FFT methods. This then restricts the frequencies to a discrete grid of frequencies up to the Nyquist frequency.

We used a 5ms bin width, giving a maximum realizable frequency of $\frac{1}{2f_s} = 100$ Hz. The lowest realizable frequency is given by $\frac{1}{T}$, where T is the total recording time. For the ON- and OFF-cells this was $\frac{1}{1500} = 0.00067$ Hz, and for the NEUTRAL-cells this was $1/960 = 0.001$ Hz and $\frac{1}{3000} = 0.00033$ Hz. For comparability of the combined frequency plot for the NEUTRAL-cells the sequences were zero-padded to 100 times the length of the shorter recording.

Hierarchical bootstrapping at the rat level was used to generate confidence intervals for the average PSD. N rats (equal to the sample number of rats) were resampled at each bootstrap iteration, using all spike trains from that rat. Per bootstrap iteration, the highest magnitude peak was then taken, and the FWHM computed. 1000 bootstrap iterations were used. Hierarchical bootstrapping helps to combat the decreased variance in the bootstrapped confidence intervals caused by correlation between spike trains measured from the same rat. Examples of this correlation are the higher likelihood of similar spectral peaks being observed, and the shadowing effect of a small number of correlated samples.

In addition to the main spectral peaks for the ON- and OFF-cells, the bootstrapped spectra also show the characteristic plateau at higher frequencies (Figure 3). This is characteristic of the PSD of a spike train; for example, Poisson neurons have a flat power spectrum corresponding to the average firing rate, with a single delta function at 0. Interestingly, there are several peaks in the NEUTRAL-cell PSD above 10 Hz. These, along with the low CV of the NEUTRAL-cells, show that they are more regular than Poisson firing neurons (CV = 1).

Finally, bootstrapping was used to compute the power spectrum for the heart rate in the same manner as for the cells. Here, there was no need to adjust for cluster sizes since each heart rate was only sampled once.

Technicalities of GP fitting

Here we discuss the benefits of using the raw binned spikes for GP fitting instead of the Gaussian kernel smoothed spike trains.

One approach to the modeling the firing rate is to convolve the spike train with a Gaussian kernel at each time point:

$$x(t) = \int d\tau \phi(\tau) s(t - \tau)$$

From this we obtain the average intensity of the single spike train. We then consider this intensity $x(t)$ to be a draw from a Gaussian process ($x \sim \mathcal{GP}$). By using a Periodic kernel, this model was able to fit well to ON- and OFF-cells, predicting their periodic behavior. However, the model leaves open the possibility of negative firing rates, and makes several false assumptions. Firstly, the model allows negative firing rates to be modeled by the GP. Secondly, since we have a constant density of points, the variance of the GP under this model does not decrease with the magnitude of $x(t)$, whereas for a more plausible model of spike trains, the lower the spike density, the lower the variance of the firing rate estimate. Finally, the GP assumes that the joint density of all points is Gaussian distributed. This assumption presents issues with overfitting in the case of spike trains, because we constructed the intensity estimate $x(t)$ using a Gaussian kernel. Therefore, there is a particular length scale at which data is well-fitted by an RBF kernel, leading to overfitting. This is not an issue when using a purely periodic kernel to model the firing rate; however, it would become an issue when modeling the trends in the firing rate with a periodic * RBF kernel, as the RBF kernel easily overfits to the data. One alternative for future study would be to use two separate RBF kernels: the first additive, modeling the short scale covariance in the firing rate induced by the Gaussian kernel, and the second kernel multiplying the periodic kernel.

However, convolving the firing rate estimate first with a kernel misses the fundamental issue with this method: spike trains are inherently noisy, and if we use a kernel to produce a continuous signal before fitting the spikes, we do not gain any new information, and remove the noise which prevents the GP from overfitting.

Therefore, a better approach is to bin the spike train. We make the assumption that the spike counts in each bin are independent, so that individual bin counts are dependent only on the value of the GP at that time point. From there, we assume that the GP is the underlying rate function for a discrete distribution (Poisson or otherwise) from which spikes are drawn. This approach deals with the fact that spike counts are integers whereas the GP models continuous functions.

Overall, the fit itself is less important than evaluating the prediction of the GP on the unseen firing rate data, as due to the noise, there will always be non-zero power at all frequencies in the training data, to which the GP can fit.

Ongoing spike train statistics

Cell Type	CV (mean ± std)	ISI (s) (mean ± std)
Neutral	0.348 ± 0.264	0.165 ± 0.250
OFF	3.699 ± 3.087	0.835 ± 1.357
ON	4.909 ± 4.319	1.248 ± 4.799

Table 2: The aggregated mean CV and ISI values of each of the three ongoing cell populations.

Bayesian Modeling Percentage Peak and Nadir Sampled Statistics

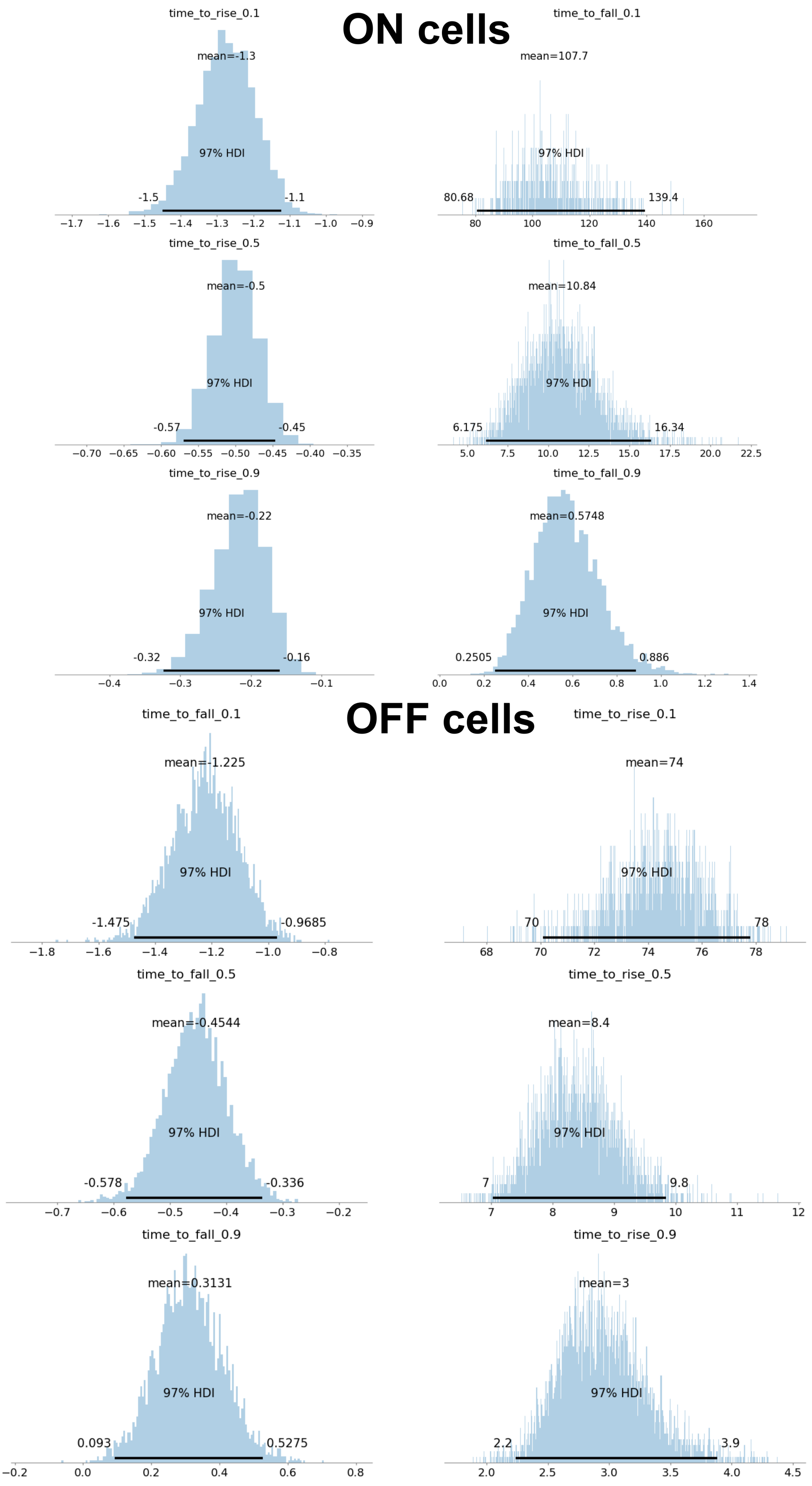

Figure 1: Posterior Distributions for the time to 10, 50 and 90% of peak for ON- cells, and 10,50 and 90% of the nadir for OFF-cells, with HDIs.

Percentile	ON cells		OFF cells	
	Rise(s)	Fall(s)	Fall(s)	Rise(s)
10%	−1.3 [−1.5, −1.1]	107.7 [80.68, 139.4]	−1.225 [−1.475, −0.9685]	74 [70, 78]
50%	−0.5 [−0.57, −0.45]	10.84 [6.175, 16.34]	−0.4544 [−0.578, −0.336]	8.4 [7, 9.8]
90%	−0.22 [−0.32, −0.16]	0.5748 [0.2505, 0.886]	0.3131 [0.093, 0.5275]	3 [2.2, 3.9]

Table 3: Mean and 97% HDI for rise and fall times in ON and OFF cells at 10%, 50%, and 90% percentiles. Rises and falls for ON-cells are measured from *base to peak*. Rises and falls for OFF-cells are measured from *base to nadir*.

Ongoing activity phase did not affect withdrawal latency

Given that ON- and OFF-cells exhibited slow, quasi-periodic fluctuations in their ongoing activity, we next asked whether the phase of these rhythms influenced behavioral responsiveness to noxious stimuli. In principle, if RVM activity modulates nociceptive gain over time, withdrawal latency might vary systematically with the predicted phase of ON- and OFF-cell activity at stimulus onset.

However, the present experiments were not designed to investigate the relationship between cell dynamics and nociceptive sensitivity. Heat stimuli were not delivered at fixed intervals but rather during periods chosen to facilitate cell classification (OFF-cells active, ON-cells silent). Moreover, contact (Peltier) and radiant heat were used in different experiments, and the number of trials for each cell was small.

Despite these constraints, we explored whether withdrawal latency correlated with the phase or predicted firing rate derived from the periodic GP model fit to each neuron's ongoing activity.

As shown in Supplementary Figure 10d,e,f, there was no systematic relationship between normalized withdrawal latency and either predicted firing rate or oscillatory phase at stimulus onset. This suggests that, under the present recording and stimulation conditions, the slow RVM oscillations did not measurably modulate reflex latency. Nevertheless, prior studies designed explicitly for this purpose have reported that nociceptive sensitivity is increased during periods of ON-cell dominance[27,32], consistent with a potential behavioral impact of these network states.

Neutral Cell PSTHS

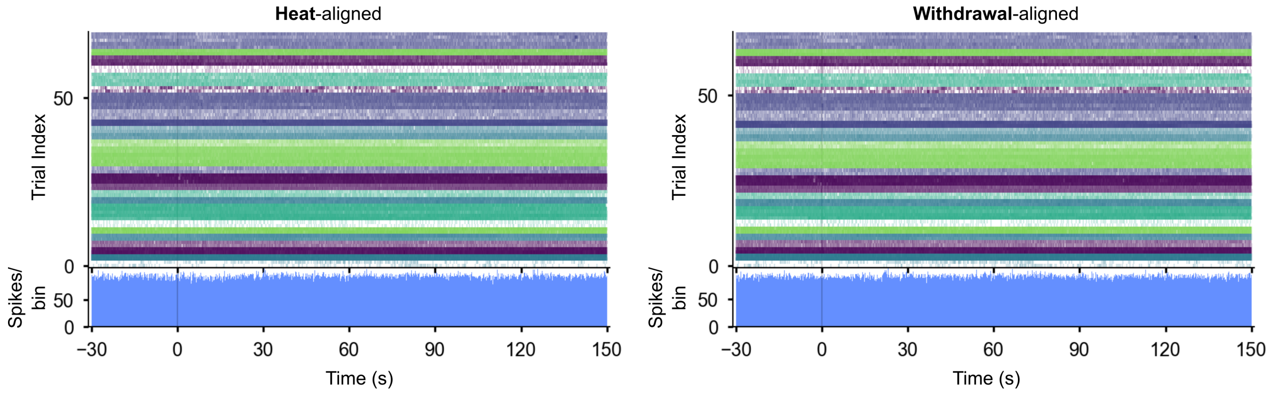

Figure 3: NEUTRAL-cells PSTHs showed no consistent modulatory effect on NEUTRAL-cells by the noxious heat or withdrawal response, nor at any time over the duration of the trial.

Bayesian Trial Model Posteriors

Throughout this section, $r_{hat} > 1.0$ indicates that the model had difficulty fitting, i.e. that the samples were either bimodal (in the case of the double exponential OFF-cell model) or that the parametric form was likely not fully correct for the data (e.g. the heat aligned ON-cell). *posterior_rate_diff* is the difference between the pre-stimulus baseline and the post-behavior recovery baseline (for the double exponential models).

ON-cells

	mean	sd	hdi_3%	hdi_97%	mcse_mean	mcse_sd	ess_bulk	ess_tail	r_hat
r_post	8.485	4.113	2.034	15.631	1.225	0.980	13.000	59.000	1.240
r_pre	41.207	0.463	40.425	42.088	0.070	0.050	49.000	799.000	1.060
k_post	65.356	21.225	26.667	83.452	10.496	8.026	6.000	33.000	1.810
k_slow	42.044	21.113	26.124	81.947	10.453	7.995	6.000	31.000	1.710
tau_post_slow	99.382	54.264	5.535	146.965	26.796	20.487	6.000	30.000	1.850
t_switch	8.623	0.080	8.481	8.769	0.016	0.011	28.000	44.000	1.100
k_pre	74.677	2.148	71.453	78.508	0.649	0.476	12.000	50.000	1.230
tau_post_fast	38.228	54.631	5.295	141.629	27.057	20.697	6.000	32.000	1.750
tau_pre	0.949	0.073	0.812	1.083	0.006	0.004	167.000	666.000	1.030

Table 4: ON-cell heat-aligned model posterior, using the double exponential model.

	mean	sd	hdi_3%	hdi_97%	mcse_mean	mcse_sd	ess_bulk	ess_tail	r_hat
r_post	35.729	2.950	30.158	40.650	0.096	0.068	1072.000	1058.000	1.000
r_pre	41.592	0.521	40.622	42.582	0.010	0.007	2816.000	2341.000	1.000
k_post	57.446	1.711	54.348	60.594	0.047	0.034	1517.000	1189.000	1.000
k_slow	40.568	3.119	34.873	46.394	0.056	0.040	3128.000	2707.000	1.000
tau_post_slow	61.252	7.868	47.781	75.855	0.259	0.190	1067.000	1101.000	1.000
t_switch	-0.176	0.040	-0.249	-0.102	0.001	0.001	1717.000	1952.000	1.000
k_pre	92.151	3.711	85.167	99.094	0.090	0.064	1705.000	2180.000	1.000
tau_post_fast	2.850	0.586	1.735	3.913	0.017	0.012	1263.000	1953.000	1.000
tau_pre	0.481	0.044	0.401	0.564	0.001	0.001	2069.000	2431.000	1.000
posterior_rate_diff	5.863	3.016	0.770	11.675	0.097	0.076	1082.000	1071.000	1.000

Table 5: ON-cell withdrawal-aligned model posterior, using the double exponential model.

	mean	sd	hdi_3%	hdi_97%	mcse_mean	mcse_sd	ess_bulk	ess_tail	r_hat
r_post	45.745	0.720	44.425	47.138	0.016	0.012	1915.000	2202.000	1.000
r_pre	41.626	0.523	40.618	42.573	0.010	0.007	2696.000	2602.000	1.000
t_switch	-0.358	0.039	-0.432	-0.288	0.001	0.001	2525.000	2271.000	1.000
k_pre	65.063	1.037	63.189	67.087	0.023	0.016	1976.000	2627.000	1.000
k_post	60.944	0.784	59.415	62.357	0.011	0.008	4686.000	2782.000	1.000
tau_post	33.677	1.389	31.114	36.308	0.033	0.023	1757.000	2224.000	1.000
tau_pre	0.463	0.054	0.362	0.561	0.001	0.001	2371.000	2113.000	1.000

Table 6: ON-cell withdrawal-aligned model posterior, using the single exponential model.

OFF-cells

	mean	sd	hdi_3%	hdi_97%	mcse_mean	mcse_sd	ess_bulk	ess_tail	r_hat
a	37.254	0.329	36.631	37.861	0.005	0.003	4869.000	2722.000	1.000
b	9.143	0.571	8.044	10.162	0.017	0.012	1148.000	1321.000	1.000
c	18.644	0.285	18.088	19.157	0.006	0.004	2237.000	2421.000	1.000
k	1.858	0.192	1.514	2.235	0.005	0.003	1705.000	2176.000	1.000
k2	0.264	0.036	0.199	0.326	0.001	0.001	1907.000	1751.000	1.000
tau_post	3.850	0.504	2.941	4.805	0.012	0.008	1907.000	1751.000	1.000
k3	0.063	0.005	0.053	0.073	0.000	0.000	2121.000	2099.000	1.000
t_switch	12.381	0.475	11.539	13.275	0.013	0.009	1423.000	1734.000	1.000
u0	9.192	0.531	8.206	10.158	0.015	0.011	1189.000	1548.000	1.000

Table 7: OFF-cell heat-aligned model posterior, using the exponential+linear model. $\tau_{post} = 1/k_2$.

	mean	sd	hdi_3%	hdi_97%	mcse_mean	mcse_sd	ess_bulk	ess_tail	r_hat
a	38.849	0.465	38.021	39.784	0.007	0.005	4847.000	2878.000	1.000
b	8.208	0.566	7.193	9.289	0.017	0.012	1058.000	1439.000	1.000
c	18.259	0.270	17.766	18.773	0.006	0.004	2366.000	2465.000	1.000
k	2.895	0.368	2.280	3.608	0.009	0.006	1845.000	2107.000	1.000
k2	0.241	0.029	0.190	0.296	0.001	0.000	2092.000	1690.000	1.000
tau_post	4.206	0.487	3.250	5.081	0.011	0.008	2092.000	1690.000	1.000
k3	0.063	0.005	0.055	0.072	0.000	0.000	2205.000	2183.000	1.000
t_switch	2.234	0.396	1.527	3.004	0.012	0.009	1223.000	1260.000	1.000
u0	8.241	0.537	7.288	9.284	0.016	0.011	1105.000	1561.000	1.000

Table 8: OFF-cell withdrawal-aligned model posterior, using the exponential+linear model. $\tau_{post} = 1/k_2$.

	mean	sd	hdi_3%	hdi_97%	mcse_mean	mcse_sd	ess_bulk	ess_tail	r_hat
a	39.001	0.466	38.125	39.881	0.007	0.005	4941.000	3012.000	1.000
b	7.940	0.591	6.826	8.997	0.018	0.013	1105.000	1186.000	1.000
c	18.415	1.452	16.691	21.598	0.436	0.317	20.000	31.000	1.300
d	19.558	2.183	16.469	24.190	0.467	0.334	24.000	1917.000	1.120
k	2.856	0.378	2.192	3.585	0.009	0.006	1843.000	1700.000	1.000
k2	0.184	0.105	0.004	0.285	0.051	0.039	7.000	33.000	1.530
k3	0.066	0.102	0.003	0.258	0.050	0.039	7.000	30.000	1.530
tau_post_fast	37.600	60.748	2.924	161.821	28.945	22.000	7.000	33.000	1.530
tau_post_slow	107.018	67.666	2.922	197.571	29.842	22.430	7.000	30.000	1.530
t_switch	2.081	0.372	1.356	2.759	0.010	0.008	1383.000	1250.000	1.000
u0	7.997	0.538	6.984	8.995	0.016	0.011	1142.000	1327.000	1.000
posterior_rate_diff	9.026	2.282	4.626	12.896	0.052	0.037	1916.000	2327.000	1.000

Table 9: OFF-cell withdrawal-aligned model posterior, using the double exponential model.

Permutation tests

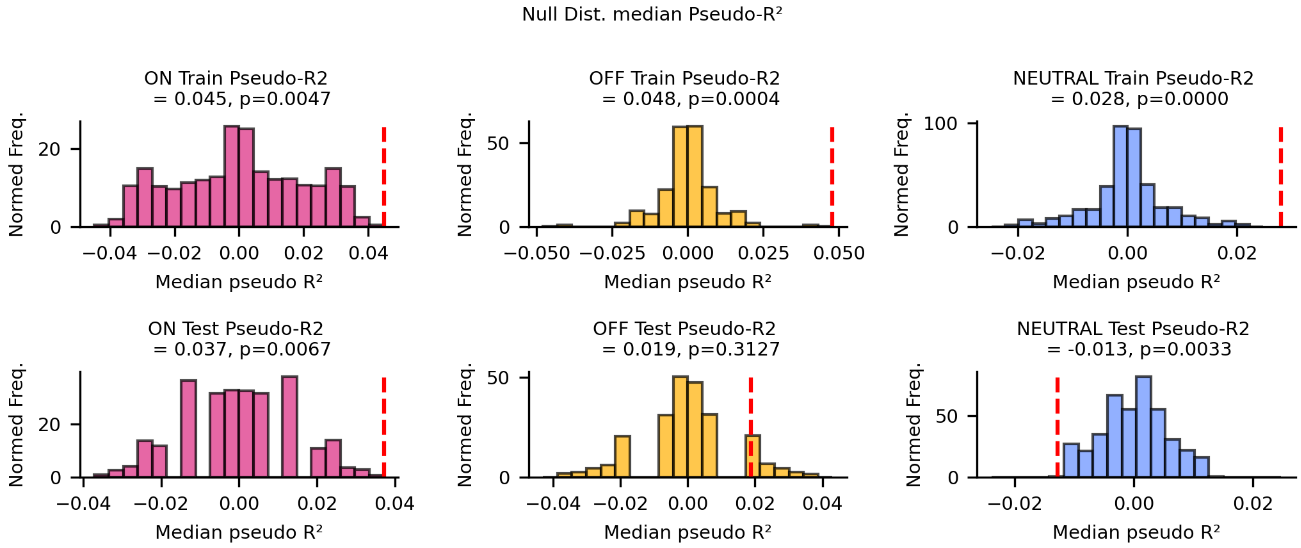

Figure 4: Two sided T-test for train and test R^2 (sign flipping permutation within animals, $N_{perm} = 10000$).

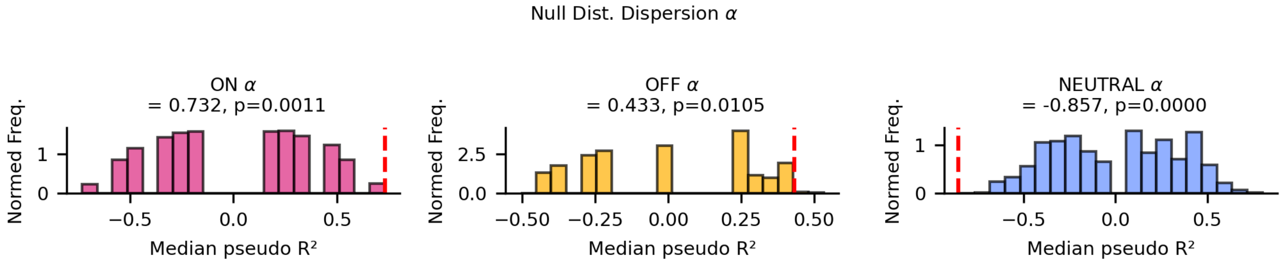

Figure 5: Two sided T-test for dispersion parameter α (sign flipping permutation within animals, $N_{perm} = 10000$).

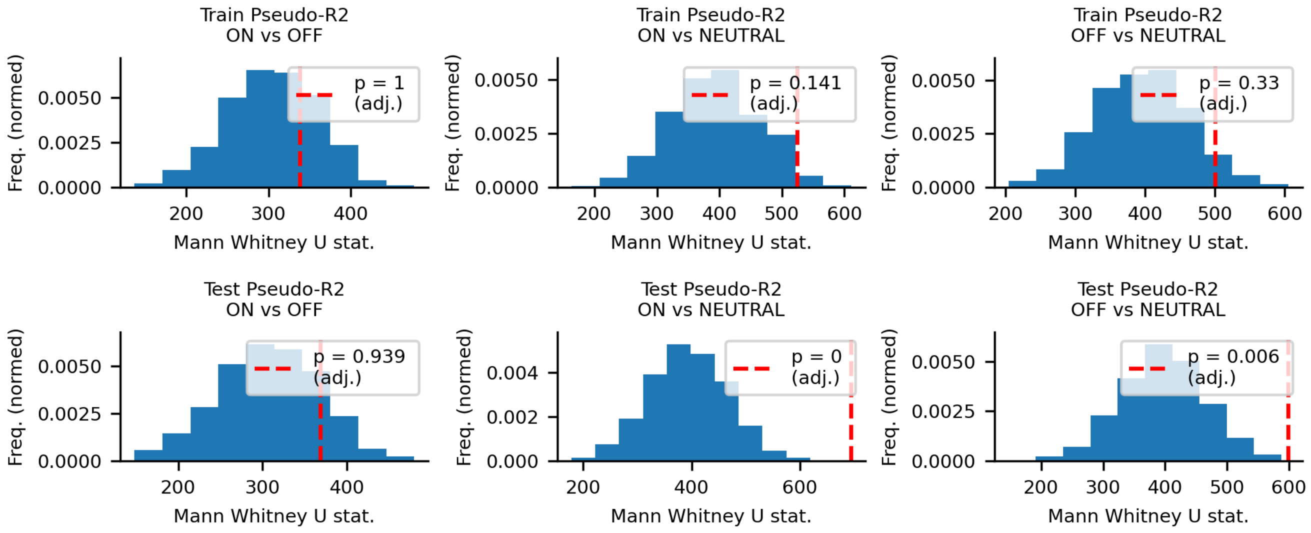

Figure 6: Mann Whitney U tests, Bonferroni corrected for multiple comparisons (sign flipping permutation within animals, $N_{perm} = 1000$).

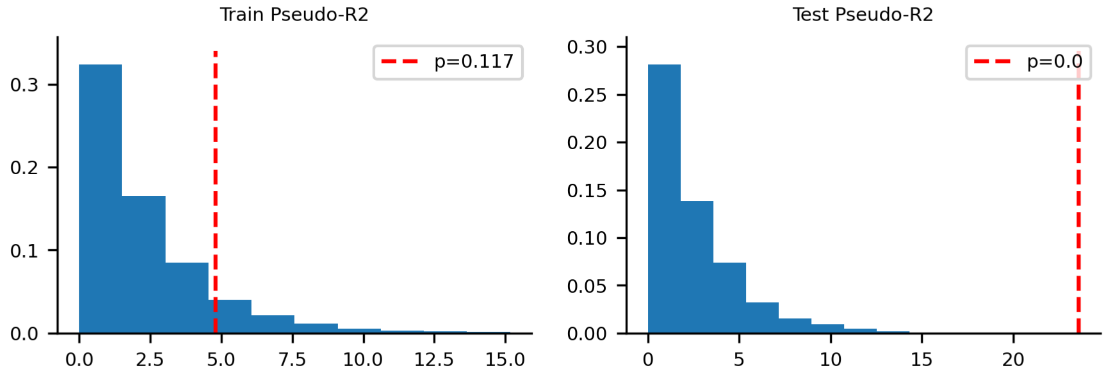

Figure 7: Kruskal-Wallis tests for differences in group medians (sign flipping permutation within animals, $N_{perm} = 1000$). Significant differences were observed at test time but not at train time.

Full Dataset Details

Protocol	Animals	# ON-, OFF-, NEUT	Recording Details	Analyses
Evoked	35	45, 35, 4	5 min. spaced trials	Piecewise Regression
Evoked/Ongoing	18	0,0,20	5 min. spaced trials	Piecewise Reg., GP fit, & PSD.
Ongoing	50	25,25,12	Ongoing (trial bookends)	Piecewise Reg, GP fit, PSD.
Totals	103	166 (70, 60,36)		

Table 10: Breakdown of data by cells recorded, trial type, and analysis performed.

Null Bayesian Models

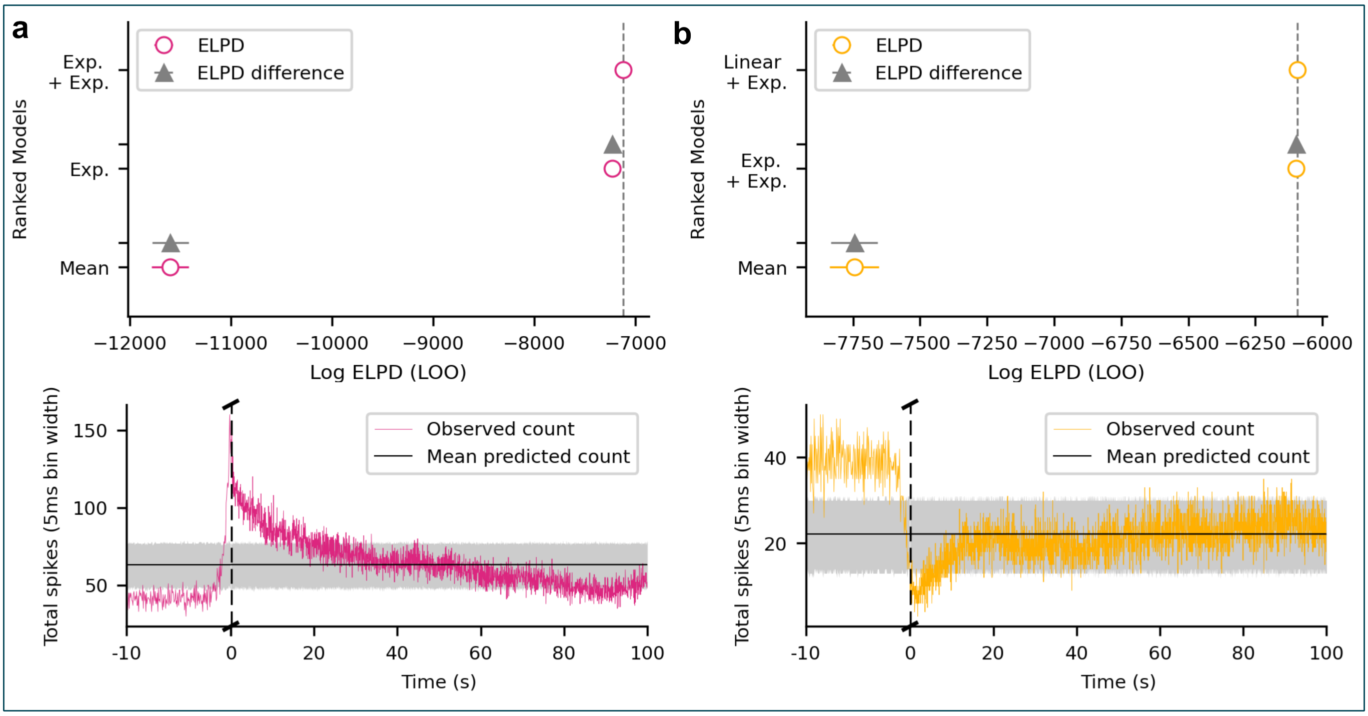

Figure 8: **a**, The null model comparison (top) and fit (bottom) for ON-cells. **b**, the null model comparison (top) and fit (bottom) for OFF-cells. Both null models clearly performed worse than either of the parametric fits.

GP Residuals

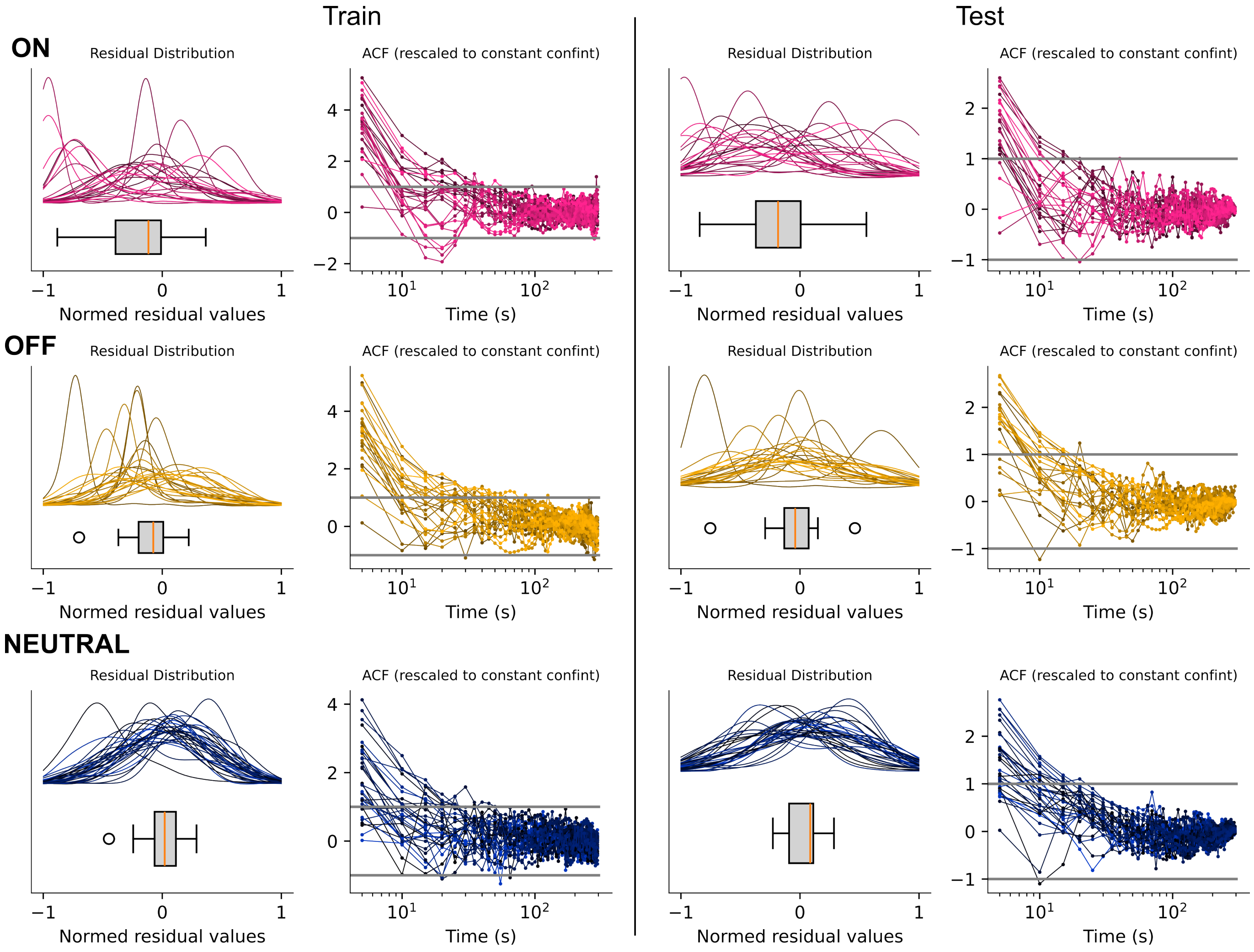

Figure 9: Gaussian process residuals for each cell. Residuals were normalized between -1 and 1 for combined plotting. Boxplots show the distribution of the mean of each residual. Fliers (circled) are Q3+1.5 IQR or Q1-1.5 IQR.

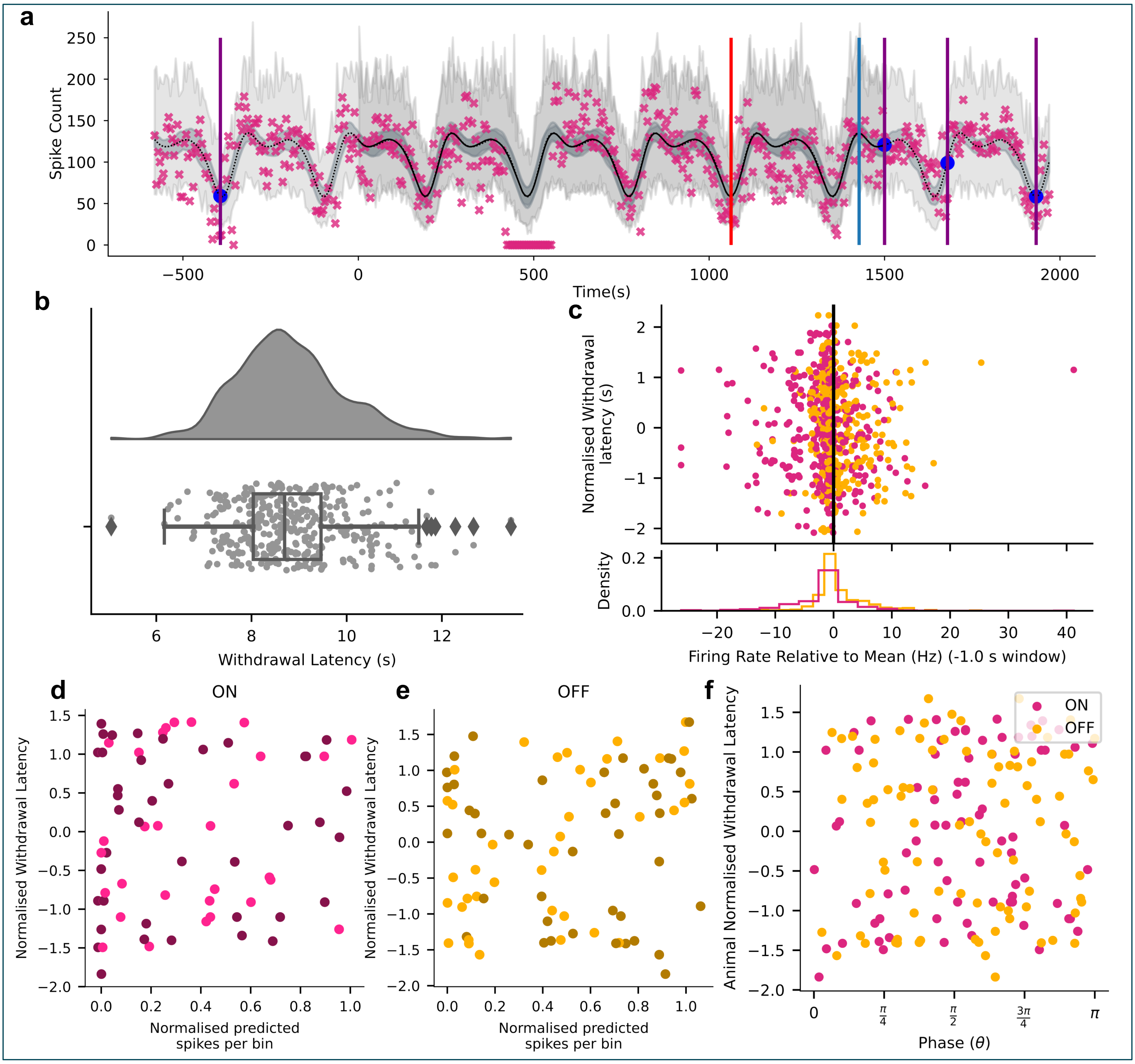

Figure 10: **a**, Example ON-cell GP fit from 0 to 1500 seconds, with extrapolated firing rate to trial times, indicated with dark blue circles. Minimum firing rate (red) and maximum firing rate (turquoise) were used to calculate the GP phase at each trial time. **b**, The distribution of withdrawal latencies across all trials and animals. **c**, The mean firing rate relative to mean of each cell before a trial, and the corresponding withdrawal latency. There was no visible effect of pre-heat firing rate on withdrawal latency, although a slight separation between ON (pink) and OFF cells (yellow) along the x-axis can be seen, potentially due to the difficulty of observing an OFF-cell if it was not already firing, and vice-versa for ON-cells. **d**, The spike count per bin predicted by the GP, normalized between the max and minimum spike count per GP fit, showed no visible correlation with withdrawal latency normalized per cell for ON-cells (**e**) or OFF-cells (**f**). The predicted GP phase showed no visible correlation with normalized withdrawal latency.

Phase Analysis

Histology Images

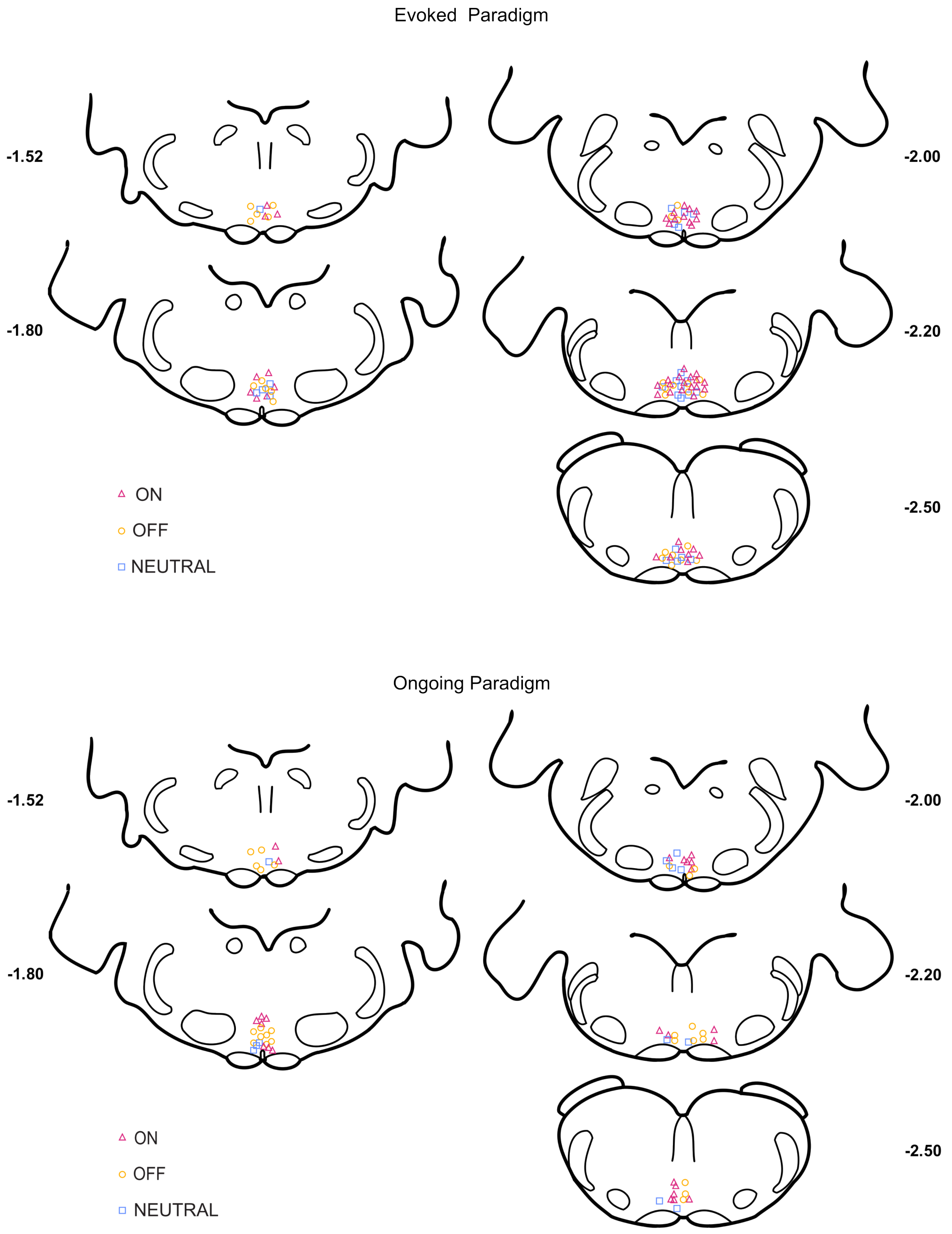

Figure 11: Reconstructed locations of all cells recorded from the RVM in the evoked and ongoing paradigms.
